# Damage-Induced Calcium Signaling and Reactive Oxygen Species Mediate Macrophage Activation in Zebrafish

**DOI:** 10.3389/fimmu.2021.636585

**Published:** 2021-03-26

**Authors:** Tamara Sipka, Romain Peroceschi, Rahma Hassan-Abdi, Martin Groß, Felix Ellett, Christina Begon-Pescia, Catherine Gonzalez, Georges Lutfalla, Mai Nguyen-Chi

**Affiliations:** ^1^ LPHI, Univ Montpellier, CNRS, Montpellier, France; ^2^ Bateson Centre and Department of Infection and Immunity, University of Sheffield, Sheffield, United Kingdom; ^3^ BioMEMS Resource Center, Center for Engineering in Medicine and Surgery, Massachusetts General Hospital and Harvard Medical School, Boston, MA, United States

**Keywords:** macrophage activation, wound healing, calcium, reactive oxygen species, zebrafish

## Abstract

Immediately after a wound, macrophages are activated and change their phenotypes in reaction to danger signals released from the damaged tissues. The cues that contribute to macrophage activation after wounding *in vivo* are still poorly understood. Calcium signaling and Reactive Oxygen Species (ROS), mainly hydrogen peroxide, are conserved early wound signals that emanate from the wound and guide neutrophils within tissues up to the wound. However, the role of these signals in the recruitment and the activation of macrophages is elusive. Here we used the transparent zebrafish larva as a tractable vertebrate system to decipher the signaling cascade necessary for macrophage recruitment and activation after the injury of the caudal fin fold. By using transgenic reporter lines to track pro-inflammatory activated macrophages combined with high-resolutive microscopy, we tested the role of Ca²⁺ and ROS signaling in macrophage activation. By inhibiting intracellular Ca²⁺ released from the ER stores, we showed that macrophage recruitment and activation towards pro-inflammatory phenotypes are impaired. By contrast, ROS are only necessary for macrophage activation independently on calcium. Using genetic depletion of neutrophils, we showed that neutrophils are not essential for macrophage recruitment and activation. Finally, we identified Src family kinases, Lyn and Yrk and NF-κB as key regulators of macrophage activation *in vivo*, with Lyn and ROS presumably acting in the same signaling pathway. This study describes a molecular mechanism by which early wound signals drive macrophage polarization and suggests unique therapeutic targets to control macrophage activity during diseases.

## Introduction

Tissue wounding induces an immediate response necessary to restore epithelial barriers and stop pathogen entry. This includes the rapid recruitment of the leukocytes to the injured tissue, including neutrophils and macrophages which are activated by danger signals and initiate inflammation. Macrophages remain associated to the wound until the tissue heals completely [reviewed in (1, 2)] and beside their function in the defence against pathogens and the elimination of cell debris, they play crucial roles in orchestrating the different steps of the healing process including granulation tissue formation, epithelialization, angiogenesis, matrix deposition and remodeling (3–6). Macrophages are also instrumental for the regeneration steps in regenerative species (7–10). Hence controlling macrophage function appears as an attractive approach to promote tissue repair in patients with trauma or degenerative disorders.

These different functions played by macrophages during wound healing can be explained by the remarkable plasticity of macrophages that are able to acquire different phenotypes based on the environmental cues that have been received. This process is called macrophage activation or polarization. Indeed, macrophage phenotypes associated to wounds have been studied in the past, mostly in mouse models with skin injury, liver injury or muscle injury (11–13). It has been shown that upon infiltration, during the early stages of inflammation, a first wave of macrophages polarizes towards pro-inflammatory M1-like phenotype, showing increased bactericidal activity, production of pro-inflammatory cytokines like TNF-α and IL-6 and removal of cellular debris (4, 12). Then, during the resolution phase, changing environmental cues trigger macrophages to shift to an anti-inflammatory M2-like phenotype and secrete anti-inflammatory cytokines like TGF-β1 and IL-10. These M2-like macrophages play an important role in tissue repair including re-epithelization, re-vascularization and fibroblast regeneration as well as in the resolution of inflammation (4, 12, 14, 15). Further, changes in macrophage phenotypes (M1/M2) are associated to chronic wounds (16). Other classifications of macrophages have been proposed: macrophages may be classified as pro-inflammatory, wound-healing or regulatory macrophages (17). Recently this simplistic view of macrophage polarization has been challenged by the discovery that macrophage phenotypes encompass a larger array of activation states and profiles, beyond M1 and M2, *in vitro* and *in vivo*, suggesting that more than 2 macrophage phenotypes are involved in the wound healing process (18–20).

Many signals were shown to stimulate the immune system including macrophages (21) and are thought to be released in response to tissue damage. These signals, commonly called damage-associated molecular patterns (DAMPs), are endogenous molecules that are released during cellular injury by dying cells or degraded extracellular matrix. Very diverse in nature, DAMPs include intracellular proteins as the chromatin-associated protein high-mobility group box 1 (HMGB1), purine metabolites as ATP, amino acids, bioactive lipids leaking from damaged cells and extracellular matrix fragments, as Hyaluronan. They are recognized by pattern recognition receptors (PRRs) or DAMP specific receptors like RAGE or CD44 which, upon ligand binding, activate conserved downstream signal transduction pathways, such as the Nuclear factor-κB (NF-κB) pathway that ultimately lead to the transcription of pro-inflammatory cytokines and chemokines involved in inflammation (22). While most of these DAMPs have been identified based on their ability to stimulate inflammatory response *in vitro*, it is unclear what real contribution these individual DAMPs make in terms of macrophage recruitment and polarization during wound healing *in vivo*.

During the last years, increasing number of studies has described the role of early wound signals in leukocyte recruitment and tissue repair. These signals, highly conserved through evolution, act within minutes as danger signals for the immune system (23). Upon injury, a gradient of Reactive Oxygen Species (ROS), the hydrogen peroxide (H_2_O_2_), emanates at the wound margin and acts as a chemotaxis signal to trigger the recruitment of neutrophils (24) in the tail fin of the zebrafish larvae. This H_2_O_2_ is produced by the NADPH oxidase DUOX (orthologue of DUOX1) in epithelial cells of the wound and diffuses through the tissues (24). Similarly DUOX-mediated H_2_O_2_ gradient is also detected in the injured epidermis of *Drosophila melanogaster* where it can trigger the recruitment of hemocytes (equivalent to leukocytes in vertebrates) (25). In the zebrafish, it has been proposed that H_2_O_2_ drives neutrophil attraction to the wound by entering the cytoplasm and inducing the oxidation of Lyn, the Src family kinase (SFK) which acts as a redox-sensor in neutrophils (26). In addition, in the same *in vivo* model, NF-κB, a master regulator of inflammation, has been suggested to be regulated by H_2_O_2_ as redox-sensitive transcription factor since DUOX-mediated H_2_O_2_ activates NF-κB signaling in the fin fold after an injury (27). Besides H_2_O_2_, other early wound signals are the Ca^2+^ waves which correspond to transient elevations of Ca^2+^ in the cytosol of the cells at the wound margin which spread from the site of injury in the skin. In *Drosophila* and zebrafish model, calcium waves initiate immune cell migration toward the wound through DUOX activation and H_2_O_2_ release (25, 27). Contradictorily, another study in zebrafish suggests that H_2_O_2_ and calcium signaling are both required for late regenerative steps of the fin fold, but act independently (28). Finally, after laser-induced brain damage in zebrafish larva, rapid Ca^2+^ waves define which microglia, the resident population of macrophages in the brain, move to the injury site. Interestingly, in this system microglia recruitment is not dependent on H_2_O_2_ (29). The exact role of H_2_O_2_ and calcium signaling in macrophage recruitment to the wound are still unclear. In addition, nothing is known about their role in the polarized activation of macrophages at the wound and the network of signaling cascade which are activated and underlie M1 macrophage activation.

Thanks to its transparency, genetic amenability and permeability to small molecules, the zebrafish larva is emerging as a tractable model system to dissect the role of wound signals after an injury. Previously, others and us showed that following caudal fin fold amputation, zebrafish macrophages are recruited and acquire different functional phenotypes, very similar to that of mammals (8, 9, 30–32). After injury, macrophages adopt a pro-inflammatory (M1-like) phenotype expressing pro-inflammatory cytokines such as *tnfa*, *tnfb*, *il1b* and *il6* (8, 32–35). While repair proceeds, macrophages switch toward non-inflammatory phenotypes expressing *cxcr4*, *ccr2* and *tgfb1* (34). During regenerative stages, pro-regenerative phenotypes were also described and were characterised by Wilms Tumor 1b expression (36). In the present study, we used fluorescent reporter zebrafish lines to track macrophage M1-like activation after injury of the fin fold and we dissected the role of ROS and calcium signaling in macrophage recruitment and polarization. In addition, we identified potential regulators, present in macrophages, which are enrolled in pro-inflammatory M1-like activation.

## Material and Methods

### Ethics Statement

Animal experimentation procedures were carried out by following the 3Rs - Replacement, Reduction and Refinement principles according to the European Union guidelines for handling of laboratory animals (http://ec.europa.eu/environment/chemicals/lab_animals/home_en.htm) and were approved by the Comité d’Ethique pour l’Expérimentation Animale under reference CEEA-LR- B4-172-37 and APAFIS#5737-2016061511212601 v3.

### Fish Husbandry

Zebrafish (*Danio rerio*) maintenance, staging and husbandry were performed at the fish facility of the University of Montpellier as described previously (37). Strains used were golden and AB WT strains and following transgenic lines: *Tg(mfap4:mCherry-F)ump6Tg* referred as *Tg(mfap4:mCherry-F)* (38) and *Tg(mpeg1:GFP-caax)sh425Tg* (referred as *Tg(mpeg1:GFP-caax)* (39)were used to visualize macrophages. *Tg(mpx:eGFP)i114*, referred as *Tg(mpx:GFP)* was used to visualize neutrophils (40). *Tg(tnfa:GFP-F)ump5Tg*, referred as *Tg(tnfa:GFP-F)* was used to visualize cells expressing Tnfa (34). *Tg(il1b9:GFP-F)ump3tg*, referred as *Tg(il1b:GFP-F)* was used to visualize cells expressing Il-1b (41). *Tg(mpx:Gal4VP16)i222* (42) and *Tg(UAS-E1b:nfsB-mCherry)i149* (43), referred as *Tg(mpx:Gal4/UAS:nfsB-mCherry)* were used to ablate neutrophils. *Tg(6xNFκB:EGFP)nc1*, referred as *Tg(NFκB-RE:eGFP)* was used to visualize NF-κB activation (44). All transgenic lines are maintained in a mix genetic background of AB and golden, with exception of the *Tg(NFκB-RE:eGFP)* which is in a Casper genetic background. Embryos were obtained from pairs of adult fishes by natural spawning and raised at 28.5°C in the embryo water. For experiments, embryos and larvae were staged according to (45) and used from 2 to 3 days post-fertilization (dpf).

### Fin Fold Transection

The caudal fin fold transection was performed at the larval stage 3 dpf. Larvae were anesthetized with 80-100 μg/mL buffered tricaine solution. The amputation was performed with scalpel, at the limit of notochord posterior end. After the amputation, larvae were placed in tricaine-free zebrafish water containing or not the chemicals or enzyme, and incubated at 28.5°C until imaging.

### Drug Treatments and Morpholino Injections

**VAS2870** (Sigma-Aldrich SML0273) stock was prepared in DMSO at 15 mM. 3 dpf larvae were injected in the yolk with 5 nl of 20 µM VAS2870 diluted in miliQ water, or with 5 nl of DMSO diluted in the same way, 20 minutes (min) before transection of the caudal fin fold.

**Apocynin** (Santa Cruz, CAS498-02-2) was dissolved at 100 mM in DMSO stock solution. One hour before the tail transection, 3 dpf larvae were placed in fish water containing 250 µM Apocynin, or the same percentage of DMSO as a control (0,25%). After the fin fold transection, the larvae were incubated in Apocynin or DMSO at 28.5°C for 6 hours, and imaged at 6 hours post-amputation (hpA).

**Thapsigargin** (Sigma-Aldrich, T9033) stock was prepared in DMSO at 2 mM. To confirm the bioactivity of thapsigargin at concentration of 1 μM, the WT strain was injected with 2-3 nL of 60 ng/μL of pGFP_CMV : GCaMP6s plasmid (Addgene, ID: 40753) at one cell-stage. GFP**^+^** larvae were selected at 3 dpf and treated with 1 μM Thapsigargin or same volume of DMSO for 6 hours. The fin fold was then injured by scalpel and imaged immediately after the cut to follow the oscillation of Ca^2+^ concentration at the wound.

To test macrophage recruitment and activation, immediately after the fin fold transection 3 dpf larvae were incubated at 28.5°C in fish water containing 1 μM Thapsigargin or same concentration of DMSO (0,05%). After 1 h thapsigargin was removed and replaced with fresh zebrafish water and imaged at 6hpA.

**Apyrase** (from potatoes, Sigma-Aldrich, A6535-100UN) stock was prepared in 30 mM Hepes buffer, pH=7.6 to obtain 100 U/mL. Immediately after the fin fold transection, larvae were placed in fish water containing or not 10 U/mL Apyrase solution as previously described (27). Larvae were incubated at 28.5°C in the enzyme solution, protected from light, until imaging at 6 hpA.

**Bay11-7082** (Sigma-Aldrich, B5556) stock was prepared in DMSO at 10 μg/μL. Immediately after the tail transection, 3 dpf larvae were placed in fish water containing 1 μg/mL Bay11-7082 solution, or DMSO as a control (0.01%). Larvae were incubated for 1.5 hour at 28.5°C, after which the drug was replaced with fresh zebrafish water until imaging.

**PP2** (Sigma-Aldrich, P0042) stock was prepared in DMSO at 10 mM. 3 dpf larvae were incubated immediately after the fin fold transection in 20 μM PP2 (Sigma-Aldrich, P0042) or DMSO (0,2%), as previously described (46), during 6 h at 28.5°C, after which drug was washed and larvae were imaged.

**H_2_O_2_** (Sigma Aldrich H1009) was diluted at 2 mM in fish water. Immediately after the tail transection, 3 dpf larvae were placed in 2 mM H2O2 solution, or fish water only as a control. Larvae were incubated for 6 hours at 28.5°C, and imaged at 6 hpA.

### Morpholino Injections

To knock down translation of P47**^phox^**, the antisense oligonucleotide morpholino (5’ CGGCGAGATGAAGTGTGTGAGCGAG 3’), overlapping the AUG start codon was used (Phan et al., 2018). 3 nL of 0.25 mM MO*p47* ***^phox^*** were injected at one-cell stage. To knock down SFK Lyn, the splicing morpholino MO*Lyn* (5’ GAGTCTGTATTTCAGTACCATTAGC 3’) targeting the Exon9-Intron9/10 site was used (Yoo et al., 2011). 2 nL of 0.5 mM morpholino were injected at one-cell stage. To knock down SFK Yrk, the splicing morpholino MO*yrk* (5’ CAATAACTGCACAAACGCACCTTTA 3’) targeting Exon6-Intron6/7 site was used (Tauzin et al., 2014). 2 nL of 0.25 mM or 0.5 mM morpholino were injected at one-cell stage. For all MO injections, Control morpholino (standard control from Gene Tools, 5’ CCTCTTACCTCAGTTACAATTTATA 3’) was injected in the same corresponding amount at one cell-stage. The efficiency of *yrk and lyn* morpholinos was validated by RT-PCR (see following sub-section) and sequencing of the PCR products. For MO*p47^phox^* the efficiency was validated by checking the NADPH oxidase activity with DHE staining (see larva staining sub-section).

### RT-PCR Analysis

For efficiency verification of the splicing morpholinos MO*lyn* and MO*yrk*, RNA was extracted from 3 dpf larvae injected with MO*lyn*, MO*yrk* or MO*ctrl*, using NucleoSpin (MACHEREY-NAGEL) and RT–PCR (invitrogen) was performed. Primers used are the following: zlyn forward: 5’ CGAAAGCTGGATAAAGCATGCG 3’ and zlyn reverse 5’ CTGCTCTCAGGTCTCGGTGG 3’ (46); zef1a forward 5’ TTCTGTTACCTGGCAAAGGG 3’ and zef1a reverse 5’ TTCAGTTTGTCCAACACCCA 3’; zyrk forward: 5’ ACGTCCTCCTGGACTCACAG 3’ and zyrk reverse 5’ AATCAGCTCCGTCAACAGGA 3’.

### Larva Staining

Decrease of Reactive Oxygen Species production was detected by CellROX Green Reagent (Invitrogen, C10444) staining. CellROX stock solution (2.5 mM) or same volume of DMSO were added to the fish water to reach the concentration of 10 µM. Larvae were incubated 1 hour before the fin fold amputation at 28.5°C, protected from light. Immediately after the fin fold transection, larvae were placed back to the CellROX or same concentration of DMSO solution during 20 min and then imaged by epi-fluorescence microscope. To detect ROS inside leukocytes, superoxide was detected by dihydroethidium (DHE) Reagent (Sigma-Aldrich, D7008-10MG) staining. DHE stock solution (15 mM) was added to the fish medium to reach the concentration of 3 µM. *Tg(mpx:GFP)* embryos were first injected with p47^phox^ morpholino or control morpholino at one-cell stage, later, at 2 dpf, larvae were infected with *Escherichia coli* expressing GFP and were incubated at 2 hpi with DHE for 30 min at 28.5°C, then washed 2 times in fish water before imaging using confocal Spinning Disk microscopy (excitation/emission 532/605 nm).

The increase of intracellular calcium concentration at the wound margin was detected by Fluo-3-AM (Sigma-Aldrich, 121714-22-5) staining. Larvae were kept in fish water supplemented with 1-phenyl-2-thiourea (PTU) from 24 hours post-fertilization (hpf) to 3 dpf. Fluo-3-AM stock solution (1 mM) or same volume of DMSO were added to the fish medium to reach the concentration of 10 µM. Larvae were incubated 1 hour before the fin fold amputation at 28.5°C, protected from light. Immediately after the fin fold transection, larvae were imaged by epi-fluorescence microscope.

### Neutrophil Depletion

For neutrophil depletion, *Tg(mpx:Gal4/UAS:nfsB-mCherry)* larvae were placed in fish water containing freshly prepared Metronidazole (Sigma-Aldrich, M3761) at 10mM and 0.1% DMSO at 2 dpf. Treatment with 0.1% DMSO was used as a control, as well as nfsB-negative siblings treated with Metronidazole. Effect on neutrophil and macrophage population and macrophage activation was analysed at 24 hours post-treatment (hpT).

### Imaging of Living Zebrafish Larvae

Larvae were anesthetized with 100 μg/mL buffered tricaine and mounted in 1% low-melting point agarose as previously described (41). Epi-fluorescence microscopy was performed using a MVX10 Olympus microscope (MVPLAPO 1X objective; XC50 camera). Confocal microscopy was performed on ZEISS LSM880 FastAiryscan, using 20X/0.8 objective, plan apochromat equipped with DIC for transmission images, resolution at 512x512 pixels. The wavelength were respectively 488nm (Argon Laser) and 561nm (DSSP Laser) for excitation. Detection was selected at 505-550nm for PMT detector and 585-620nm for GaAsP detector. The images were taken in a sequential mode by line. The 3D files generated by multi-scan acquisitions were processed by Image J. To image Ca^2+^ oscillations at the wound, we used ANDOR CSU-W1 confocal spinning disk on an inverted NIKON microscope (Ti Eclipse) with ANDOR Neo sCMOS camera (20x air/NA 0.75 objective). Image stacks for time-lapse movies were acquired at 28°C every 20 seconds (s), with z-stack of 45 μm at 3 μm intervals. To image macrophage activation in live, z-stacks of 78 μm with 3 μm intervals were acquired every 3min, in multiposition mode. The 4D files generated from time-lapse acquisitions were processed using Image J. Brightness and contrast were adjusted for maximal visibility.

### Cell Quantification

Recruited macrophages and neutrophils were counted manually from maximum projections of obtained microscopy images, using Fiji (ImageJ) software, after the brightness/contrast adjustment for better visualization. To count recruited and M1-activated macrophages, quantification area in all experiments was 270 μm from the wound edge. To count recruited neutrophils, quantification area was 200 μm from the wound edge. For the quantification of signal intensity after CellROX staining and NFκB activation, mean gray value was extracted from the area 75 μm from the wound edge. Total macrophage population in larvae was quantified by computation using Fiji as described previously (37).

### Data Analysis and Statistics

Samples were split into experimental groups by randomization. The sample size estimation and the power of the statistical test were computed using GPower. A preliminary analysis was used to determine the necessary sample size *N* of a test given *α*<0.05, power =1- *β* > 0.80 (where *α* is the probability of incorrectly rejecting *H_0_* when is in fact true and *β* is the probability of incorrectly retaining *H_0_* when it is in fact false). Then the effect size was determined. Groups include the number of independent values, and statistical analysis was done using these independent values. Graph Pad Prism 5.01 Software (San Diego, CA, USA) was used to construct graphs and analyze data in all figures. Specific statistical tests were used to evaluate the significance of differences between groups. The type of test used as well as p-value is indicated for each graph in figure legends. The number of independent experiments (biological replicates) presented is indicated in the figure legends.

## Results

### Intracellular Ca^2+^ Signaling Mediate Macrophage Recruitment and Activation

**Figure 1:**
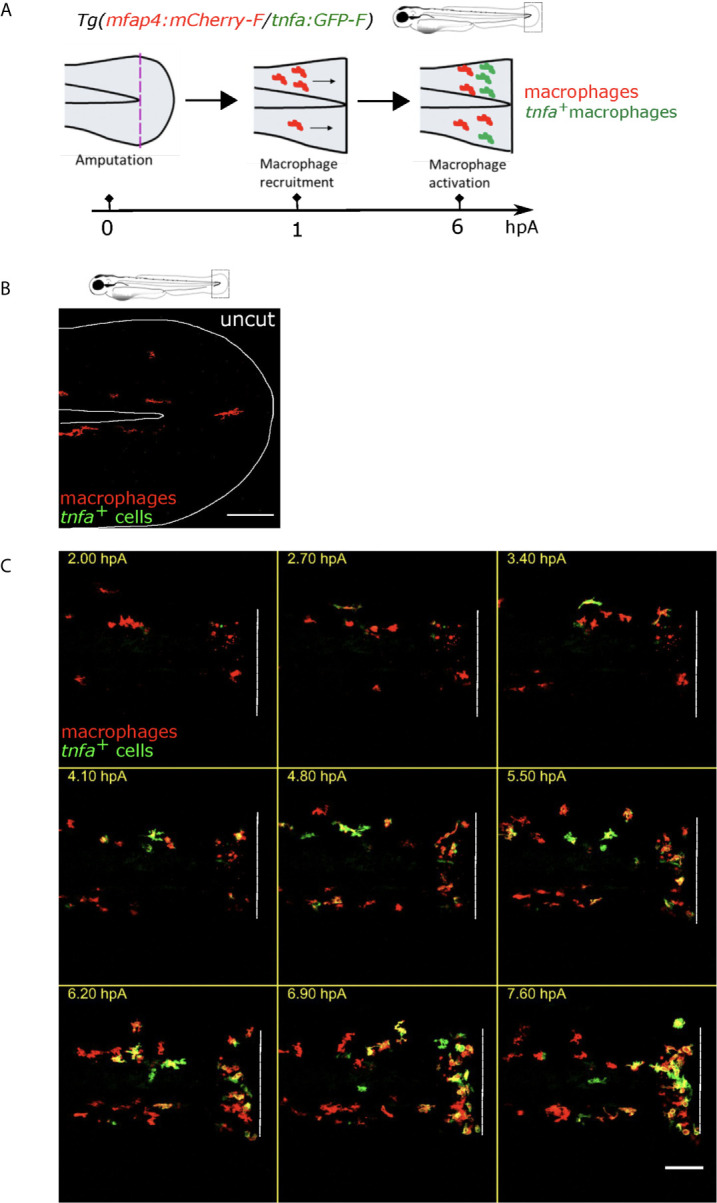
*Tg(mfap4:mCherry-F/tnfa:GFP-F)* reporter line allows imaging of macrophage M1-like activation at the wound in response to fin fold injury. **(A)** The caudal fin fold of *Tg(mfap4:mCherry-F/tnfa:GFP-F)* larvae were amputated at 3 dpf. Schedule of the recruitment and M1-like activation of macrophages at the wound after the fin fold injury. **(B)** Representative image of uncut zebrafish fin fold. Maximum projection of the overlaid fluorescence of mCherry-F (macrophages) and GFP-F (tnfa+ cells). The white line outlines the fin fold and the notochord. Scale bar: 100 μm. **(C)** Macrophage movements and activation states were imaged by confocal microscopy from 1.5 to 9.5 hours post amputation (hpA) using the *tg(mfap4:mCherry-F/tnfa:GFP-F)* line. Tail images are representative maximum projections of the overlaid fluorescences of mCherry-F (macrophages) and GFP-F (*tnfa^+^* cells), showing M1-like activation of recruited macrophages at the wound, starting from 3 hpA and increasing up to 7.6 hpA. Dashed line indicates the wound margin. Scale bar: 100 μm.

The caudal fin fold injury of the zebrafish larva induces an inflammation characterized by the recruitment and the activation of innate immune cells (32, 34, 47–49). To directly assess macrophage accumulation and pro-inflammatory (M1-like) activation, at 3 days post fertilization (dpf), we injured (amputated) the fin fold of the zebrafish reporter line *Tg(mfap4:mCherry-F/tnfa:GFP-F)* in which all macrophages express a farnesylated mCherry protein, and M1-like-activated macrophages, expressing the pro-inflammatory cytokine Tnfa, express a farnesylated GFP (**Figure 1A**). In intact fin fold, few inactivated macrophages were observed (**Figure 1B**). We performed intra-vital imaging of the tail from 1.5 to 9.5 hours post-amputation (hpA) (**Figure 1C** **, Movie S1**). This system allows us to clearly follow the recruitment and accumulation of macrophages at the wound after the fin fold amputation, and their M1-like-activation in real time and *in vivo.*

**Figure 2:**
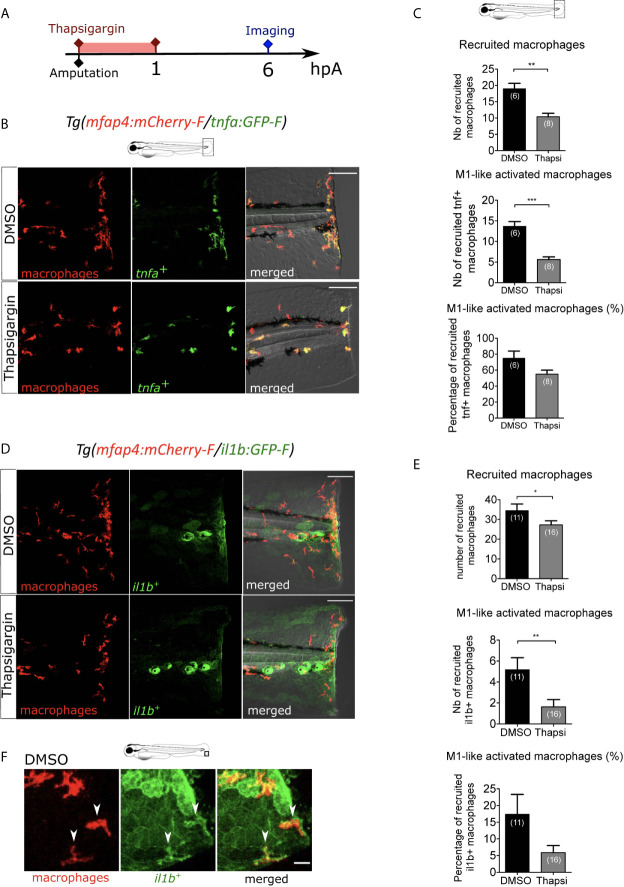
Intracellular Ca^2+^ signaling mediates macrophage recruitment and activation. **(A)** Schedule of the experiment. Immediately after the fin fold amputation at 3 dpf, *Tg(mfap4:mCherry-F/tnfa:GFP-F)* or *Tg(mfap4:mCherry/il1b:GFP)* larvae were treated with Thapsigargin or DMSO for 1 h. Thapsigargin was removed and larvae were imaged at 6 hours post amputation (hpA) using confocal microscopy. **(B)** Tail images are representative maximum projections of the fluorescence of mCherry-F (macrophages), GFP-F (*tnfa* ^+^ cells) and merged channel images of *Tg(mfap4:mCherry-F/tnfa:GFP-F)* injured larvae, after the treatment with DMSO (up) or Thapsigargin (down) at 6 hpA. Scale bars: 100 μm. **(C)** Quantification of recruited macrophages (up) and *tnfa***^+^** recruited macrophages (middle) after the DMSO and Thapsigargin treatments at 6 hpA. The lower graph represents the percentage of *tnfa***^+^** macrophages in the recruited population. Representative experiment of three independent experiments, mean ± SEM, *n_larvae_* is indicated in brackets, Mann Whitney test, two-tailed, **p<0.01, ***p<0.001. **(D)** Tail images are representative maximum projections of the fluorescence of mCherry-F (macrophages), GFP-F (*il1b* ^+^ cells) and merged channel images with brightfield of *Tg(mfap4:mCherry/il1b:GFP)* injured larvae after the treatment with DMSO (up) or Thapsigargin (down) at 6 hpA. Scale bars: 100 μm. **(E)** Quantification of recruited macrophages (up) and *il1b^+^* recruited macrophages (middle) after the DMSO and Thapsigargin treatments at 6 hpA. The lower graph represents the percentage of *il1b* ^+^ macrophages in the recruited population. Two independent experiments merged, mean ± SEM, *n_larvae_* is indicated in brackets, one-tailed t-test with Welch’s correction, *p<0.05, **p<0.01. **(F)** Representative zoomed maximum projections of the fluorescence of mCherry-F (macrophages) and GFP-F (*il1b* ^+^ cells) merge channels images in injured fin fold of *Tg(mfap4:mCherry/il1b:GFP)* at 6hpA after the treatment with DMSO. Arrow heads show the overlap between GFP and mCherry signal in macrophages. Scale bar: 20 μm.

We first focused on the role of wound-induced Ca²⁺ signaling in macrophage M1-like activation. In many animal species, rapid Ca²⁺ oscillations occur immediately after injury, and they are important for wound healing and regeneration (25, 27, 28, 50). As previously shown, cytosolic calcium elevation, detected using the calcium probe Fluo-3 AM, was observed immediately after wounding of the fin fold (**Figure S1A**). To block Ca²⁺ oscillations at the wound, we used Thapsigargin, an irreversible drug that blocks the SERCA pumps, responsible for transport of Ca²⁺ from the cytosol to the endoplasmic reticulum. To visualize the dynamics of calcium signaling, we used larvae that mosaically express the fluorescent Ca^2+^ sensor GCaMP6s, after the injection of pGFP_CMV : GCaMP6s plasmid at one-cell stage (51). Then, fin folds were wounded and imaged using spinning disk microscopy. While in DMSO treatment calcium oscillations were observed, Thapsigargin treatment efficiently blocked these oscillations (**Figures S1B-D** **, Movie S2**). To test the role of Ca²⁺ signaling on macrophage recruitment and activation, we treated *Tg(mfap4:mCherry-F/tnfa:GFP-F) larvae with* Thapsigargin. While the DMSO treatment (control) had no effect on macrophage recruitment and activation, Thapsigargin treatment significantly reduced the number of both recruited macrophages and *tnfa*-expressing macrophages at 6 hpA (**Figures 2A-C**). The decreased number of *tnfa* ^+^ macrophages was not due to less recruitment as the percentage of *tnfa* ^+^ macrophages in the recruited population was also decreased (**Figure 2C**). It was not due to a general decrease of the macrophage population since the number of total macrophages in larvae was not affected by Thapsigargin (**Figure S1E**).

The effect of Thapsigargin was further confirmed on another M1-like-reporter line, *Tg(mfap4:mCherry-F/il1b:GFP-F)*, in which macrophages expressing *il1b*, expressed both mCherry-F and GFP-F (**Figures 2D, E**). In control condition (DMSO), *il1b* is expressed at 6 hpA in different cell types at the wound including keratinocytes and macrophages. The latter are detected thanks to overlap of mCherry-F and GFP-F (*il1b* ^+^ macrophages) labeling (**Figure 2F**). As expected, after Thapsigargin treatment the number of recruited macrophages and *il1b* ^+^ macrophages is reduced compared to the control, as well as the percentage of *il1b* ^+^ macrophages in the recruited population (**Figures 2D, E**). Taken together, these results indicate that calcium signaling is involved in macrophage recruitment and pro-inflammatory M1-like activation.

### ROS Production at the Wound Mediates Macrophage Activation But Not Their Recruitment

**Figure 3:**
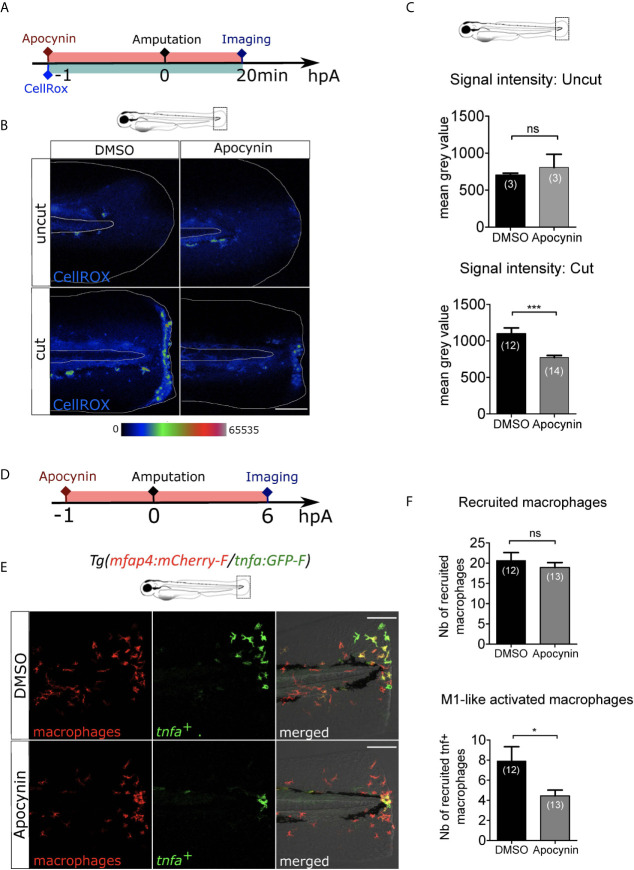
ROS release at the wound mediate macrophage activation but not recruitment. **(A)** Schedule of the experiment. 1h before the fin fold injury at 3 dpf, larvae were incubated in Apocynin or DMSO, containing CellROX solution for the detection of the ROS production. Drug and staining were both removed at 20 min pA, and larvae were immediately imaged using epi-fluorescent microscopy. **(B)** Representative images of the CellROX fluorescence in uncut or cut fin folds, after the treatment with DMSO or Apocynin at 20 min pA. Rainbow color scale was applied to images, emphasizing the differences in signal intensity. The white lines outline the fin fold and the notochord. Scale bar: 100 μm. **(C)** Quantification of signal intensity of the CellROX fluorescence by mean gray value. Representative experiment of two independent experiments, mean ± SEM, *n****_larvae_*** is indicated in brackets, upper graph: Mann Whitney test, two-tailed, ns – not significant, bottom graph: one-tailed t-test with Welch’s correction, ***p<0.001. **(D)** Schedule of the experiment. From 1 h before the fin fold amputation at 3 dpf, until 6 hpA, *Tg(mfap4:mCherry-F/tnfa:GFP-F)* larvae were incubated in Apocynin or DMSO, and then imaged at 6 hpA using confocal microscopy. **(E)** Tail images are representative maximum projections of the fluorescence of mCherry-F (macrophages), GFP-F (*tnfa* ^+^ cells) and merged channel images with brightfield after the treatment with DMSO (up) or Apocynin (down) at 6 hpA. Scale bars: 100 μm. **(F)** Quantification of recruited macrophages (up) and *tnfa***^+^** recruited macrophages (down) after DMSO and Apocynin treatments at 6 hpA. Representative experiment of two independent experiments, mean ± SEM, *n_larvae_* is indicated in brackets, one-tailed t-test with Welch’s correction, *p<0.05.

Next, we investigated whether there are other early wound signals that impact macrophage recruitment and activation. ROS, such as H_2_O_2_, are important signals produced at the wound controlling neutrophil recruitment (24). To test the role of ROS on macrophage behaviour, we treated the larvae with a general inhibitor of ROS production, Apocynin (**Figure 3A**). The efficacy of Apocynin treatment was confirmed by oxidation of the highly ROS-specific probe CellROX that fluoresce in the presence of ROS. In this assay, low signal of CellROX was detected in the uncut fin folds. As previously shown, a strong production of ROS was observed at the site of fin fold injury in DMSO condition, 20 minutes (min) post-amputation. By contrast after Apocynin treatment, ROS production was decreased at the wound showing that Apocynin efficiently blocks injury-induced ROS production (**Figures 3B, C**). Treatment of *Tg(mfap4:mCherry-F/tnfa:GFP-F)* larvae with Apocynin 1 hour before amputation did not affect the recruitment of macrophages at 6 hpA (**Figures 3D-F**). On the contrary, M1-like-activation was significantly decreased in Apocynin treated larvae, compared to controls (**Figures 3D-F**). The same effect was obtained with another ROS inhibitor, VAS2870 (**Figures S2A-F**). These matching results clearly show that ROS is an important wound signal mediating proper immune response, involved in macrophage activation, but not in their chemotaxis towards the wound.

The ROS, H_2_O_2_, is tightly regulated in time and space during wound healing (24, 52). We next tested whether an elevation of H_2_O_2_ in the zebrafish larvae alters macrophage recruitment and activation upon injury. First WT larvae were incubated in 2 mM H_2_O_2_, as performed previously (53), and with the ROS-specific probe CellROX at 3 dpf and amputated 1 hour later. Imaging of CellROX show that H_2_O_2_ treatment significantly increased ROS in the intact and injured tails (**Figures S3A-C**). Moreover, treatment of *Tg(mfap4:mCherry-F/tnfa:GFP-F)* larvae with 2 mM H_2_O_2_ led to the decrease of macrophages number at the injury site and of the percentage of tnfa^+^ macrophages at 6 hpA (**Figures S3D** and **F, G**). Further the quantification of global population of macrophages in the tails show that the global number of macrophages was not affected by the treatment (**Figure S3E**). Altogether these data show that an elevation of H_2_O_2_ impaired both the recruitment and the M1-like-activation of macrophages at the wound suggesting that narrow concentrations of H_2_O_2_ are key to macrophage function.

A number of studies point out Duox enzyme as the main source of ROS produced at the wound. The role of other NADPH enzymes such as Nox2, expressed in leukocytes, in this process is more elusive. To test the Nox2 as a potential intracellular source of ROS, we knocked down the gene coding for Nox2 subunit P47^phox^ using an antisense oligonucleotide morpholino that specifically blocks the translation of p47^phox^ (MO*p47^phox^*). The efficacy of this morpholino was confirmed by monitoring the activity of NOX activity in leukocytes. *P47^phox^* morphants and their controls were injected with *Escherichia coli* at 48 hours post-fertilization (hpf) in the muscle. At 2 hours post-infection (hpi) larvae were stained with Dihydroethidium (DHE) probe which labels superoxide $\left(O_{2}^{-}\right)$ producing cells **Figure S4A**). While in control morphants the leukocytes produced $\left(O_{2}^{-}\right)$ in response to the infection, in *p47^phox^* morphants the production of $\left(O_{2}^{-}\right)$ was decreased in leukocytes, showing that *p47^phox^* morpholino impairs NOX activity (**Figures S4B, C**). To test whether *p47^phox^* knockdown affects the function of macrophages during wound healing, *p47^phox^* morpholino or control morpholino were injected in *Tg(mfap4:mCherry-F/tnfa:GFP-F)* embryos at one-cell stage and then the morphants were amputated at 3 dpf. At 6 hpA, p47^phox^ morphants did not show any decrease in macrophage recruitment or M1-activation compared to the control morpholino (**Figures S4D-F**). This data suggested that Nox2-dependent ROS production is not involved in macrophages recruitment nor activation.

### Decrease of Recruited Neutrophils at the Wound Does Not Affect Macrophage Activation

**Figure 4:**
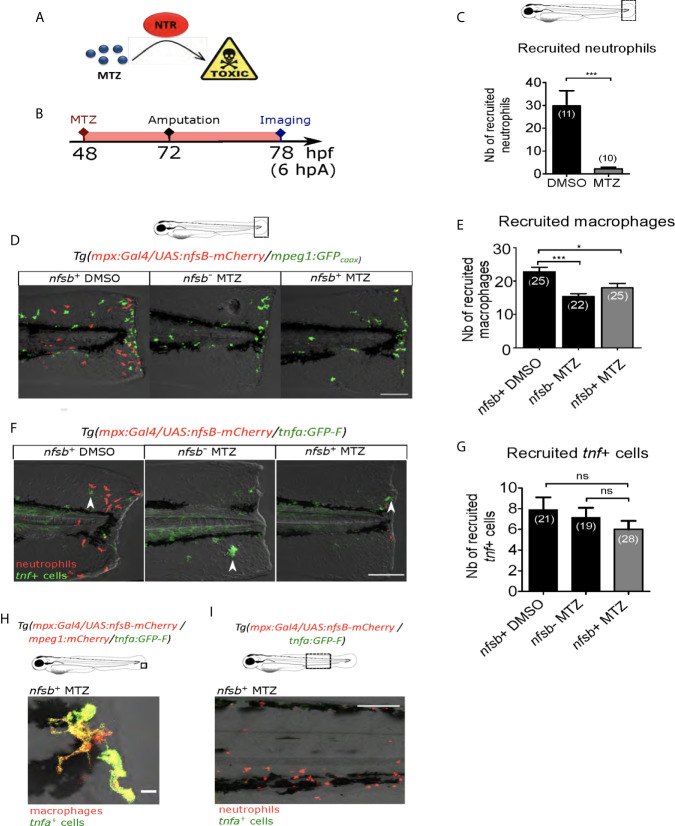
Neutrophil presence at the wound is not necessary for macrophage activation. **(A)** Mechanism of action of the prodrug metronidazole (MTZ) that is converted into a cell-autonomous toxic molecule in the presence of nitroreductase (NTR). Specific expression of NTR within neutrophils leads to the depletion and immobilization of larval neutrophils. **(B)** Schedule of the experiment. At 48 hpf *Tg(mpx:Gal4/UAS:nfsB-mCherry/tnfa:GFP-F)* or *Tg(mpx:Gal4/UAS:nfsB-mCherry/mpeg1:GFP-caax)* larvae were treated with metronidazole (MTZ) and maintained in the drug until the end of the experiment. Transgenic larvae treated with DMSO and WT siblings treated with MTZ were used as controls. At 3 dpf, fin folds were injured and larvae were imaged at 6 hpA using confocal microscopy. **(C)** Quantification of recruited neutrophils at the wound at 6 hpA, after the treatment with MTZ or DMSO. Representative experiment of three independent experiments, mean ± SEM, *n_larvae_* is indicated in brackets, one-tailed t-test with Welch’s correction, ***p<0.001. **(D)** *Tg(mpx:Gal4/UAS:nfsB-mCherry/mpeg1:GFP-caax)* treated with DMSO (*nfsb* ^+^ DMSO) or with MTZ (*nfsb* ^+^ MTZ) and WT siblings with MTZ (*nfsb* ^-^ MTZ). Tail images are representative maximum projections of the fluorescence of mCherry-F (neutrophils), GFP-caax (macrophages) and merged channel images with brightfield at 6 hpA. Scale bar: 100 μm. **(E)** Quantification of recruited macrophages at the wound at 6 hpA, after the treatment with MTZ or DMSO. Representative experiment of two independent experiments, mean ± SEM, *n_larvae_* is indicated in brackets, upper graph: one-way ANOVA, *p<0.05 ***p<0.001. **(F)** *Tg(mpx:Gal4/UAS:nfsB-mCherry/tnfa:GFP-F)* treated with DMSO (*nfsb* ^+^ DMSO) or with MTZ (*nfsb* ^+^ MTZ^+^) and WT siblings with MTZ (*nfsb* ^-^ MTZ^+^). Tail images are representative maximum projections of the fluorescence of mCherry-F (neutrophils), GFP-F (tnfa+ cells, selected by arrow heads) and merged channel images with brightfield at 6 hpA. Scale bar: 100 μm. **(G)** Quantification of recruited *tnfa***^+^** cells at the wound at 6 hpA in indicated conditions. Two independent experiments merged, mean ± SEM, *n_larvae_* is indicated in brackets, Kruskal-Wallis test, ns: not significant. **(H)** Quadruple transgenic line *Tg(mpx:Gal4/UAS:nfsB-mCherry/mfap4:mCherry/tnfa:GFP-F)* treated with MTZ and imaged at 6hpA. Image is the representative zoomed and overlaid maximum projections showing the overlap of the fluorescence of mCherry-F (macrophages) and GFP-F (*tnfa* ^+^ cells), in the absence of neutrophils at the wound. Scale bar: 10 μm. **(I)** *Tg(mpx:Gal4/UAS:nfsB-mCherry/tnfa:GFP-F)* larvae treated with MTZ and imaged at 6hpA in the CHT region. Image is a representative maximum projection of mCherry and GFP-F fluorescences overlaid with brightfield image, showing no potential *tnfa* (GFP-F^+^ cells) expressed in the vicinity of neutrophils (mCherry^+^ cells) upon MTZ treatment. Scale bar: 100 μm.

Since Ca²⁺-ROS axis is described to be important in neutrophil recruitment after the fin fold amputation of zebrafish larvae (27, 28), we tested whether the effect observed on macrophages after blocking of Ca²⁺ or ROS signaling at the wound was due to unsuccessful neutrophil recruitment and neutrophil absence at the wound. For this, we first supressed neutrophil population using the transgenic line *Tg(mpx:Gal4/UAS:nfsB-mCherry)* in which *mpx* promoter indirectly drives the expression of *nfsB* specifically in neutrophils. *nfsB* encodes an *Escherichia coli* nitroreductase (NTR) that converts the prodrug metronidazole (MTZ) into a cell-autonomous toxic agent, leading to death of the expressing cells (43) (**Figure 4A**). 48 hours post-fertilization (hpf) *Tg(mpx:Gal4/UAS:nfsB-mCherry)* larvae were treated with 10 mM of MTZ (balneation) and neutrophils were counted at 78 hpf (**Figure 4B**). DMSO treatment on transgenic larvae as well as metronidazole treatment on WT siblings were used as controls. We showed that metronidazole in transgenics efficiently reduced the number of recruited neutrophils at the wound by about 90%, compared to DMSO at 6 hpA (**Figure 4C**). First to test a possible effect of neutrophil reduction on macrophage recruitment, we crossed *Tg(mpx:Gal4/UAS:nfsB-mCherry)* with *tg(mpeg1:GFPcaax)* and performed similar treatments on transgenic larvae and their WT siblings (controls) at 48 hpf, injuring the fin fold as before at 3 dpf and then imaging at 6 hpA. Although, the number of macrophages at the wound was lower in the metronidazole-treated WTs than in DMSO-treated transgenics (32% less), the number of macrophages was not further decreased in metronidazole-treated *Tg(mpx:gal4/UAS:nfsB-mCherry/mpeg1:GFP-caax)* (**Figures 4D, E**). This suggests that metronidazole alone slightly affects macrophages recruitment but neutrophil reduction does not account for the small decrease in macrophage numbers. To test the effect of neutrophil reduction on macrophage polarization, we used triple-transgenic line *Tg(mpx:Gal4/UAS:nfsB-mCherry/tnfa:GFP)*. MTZ treatment of triple-transgenic or WT lines has no effect on the number of *tnfa*-expressing cells at the wound showing that beside neutrophil reduction macrophages were still able to activate as M1-like phenotype and express *tnfa* in the same trend like in neutrophil presence (**Figures 4F, G**). Furthermore, the *tnfa*-expressing cells at the wound were confirmed to be macrophages, as microscopy analysis of the *Tg(mpx:Gal4/UAS:nfsB-mCherry/mfap4:mCherry-F/tnfa:GFP-F)* line in the same MTZ-treatment conditions shows that *tnfa* (GFP-F) expression colocalizes with *mfap4* (mCherry-F) in the absence of neutrophils (**Figure 4H**). To verify that *tnfa* expression in macrophages was not induced by neutrophil death after MTZ treatment through a process of a global macrophage activation in the whole larvae, we imaged the Caudal Hematopoietic Tissue (CHT) region of the *Tg(mpx:Gal4/UAS:nfsB-mCherry/tnfa:GFP)* larvae 24 hours after MTZ treatment without amputation. While some remaining neutrophils were still observed in the CHT although they were less numerous that in the control, we did not observe any *tnfa^+^* cells in the vicinity (**Figure 4I**). Altogether these data suggest that both macrophage recruitment and M1-like activation are not dependent on the presence of neutrophils at the wound.

### Extracellular ATP Is Not Necessary for Macrophage Activation

**Figure 5:**
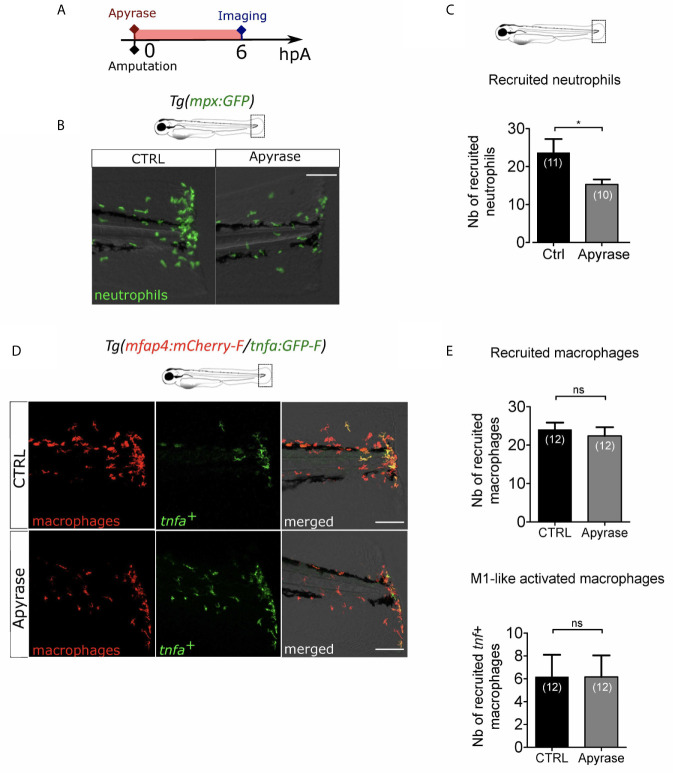
Extracellular ATP is not necessary for macrophage activation. **(A)** Schedule of the experiment. Fin folds from *Tg(mpx:GFP)* or *Tg(mfap4:mCherry-F/tnfa:GFP-F)* were injured at 3 dpf and larvae were immediately incubated in zebrafish water containing or not Apyrase and imaged at 6 hpA using epi-fluorescent or confocal microscopy. **(B)** Tail images are representative overlays of GFP fluorescence (neutrophils) and brightfield image. Images were acquired at 6 hpA by epi-fluorescent microscopy from *Tg(mpx:GFP)* larvae either untreated or treated with Apyrase. Scale bar: 100 μm. **(C)** Quantification of neutrophils recruited at the wound at 6hpA. Representative experiment of two independent experiments, mean ± SEM, *n_larvae_* is indicated in brackets, one-tailed t-test with Welch’s correction, *p<0.05. **(D)** Tail images are representative maximum projections of the fluorescence of mCherry-F (macrophages), GFP-F (*tnfa+* cells) and merged channel images with brightfield of *Tg(mfap4:mCherry-F/tnfa:GFP-F)* injured larvae after no treatment (control, up) or Apyrase treatment (down) at 6 hpA. Scale bars: 100 μm. **(E)** Quantification of recruited macrophages (up) and *tnfa***^+^** recruited macrophages (down) in controls and in Apyrase treated larvae at 6 hpA. Representative experiment of two independent experiments, mean ± SEM, *n_larvae_* is indicated in brackets, upper graph: t-test, two-tailed, ns- not significant; bottom graph: Mann Whitney test, two-tailed, ns - not significant.

Extracellular ATP is a well-described DAMP. It has been proposed as an early wound molecule that activates calcium and H_2_O_2_ signaling and triggers neutrophil recruitment at the wound (27). We thus investigated the role of extracellular ATP in the macrophage M1-like activation. We removed extracellular ATP by adding Apyrase, the ATP-hydrolyzing enzyme into the zebrafish water at 3 dpf immediately after amputation (**Figure 5A**). Firstly, we verified that Apyrase treatment of *Tg(mpx:GFP)*, a reporter line for neutrophils, decreased neutrophil recruitment as previously shown (27) (**Figures 5B, C**). Indeed, less neutrophils were present at the wound at 6hpA, thus confirming the treatment efficacy. Same procedure was repeated on *Tg(mfap4:mCherry-F/tnfa:GFP-F)* to analyse macrophage recruitment and activation. Apyrase has no detectable effect on macrophage function 6 hours post amputation, affecting neither accumulation of macrophages nor the expression of *tnfa* (**Figures 5D, E**). It is important to note that the reduced number of neutrophils at the wound upon Apyrase treatment did not correlate with any macrophage dysfunction, confirming thus that neutrophil recruitment was not necessary for macrophage attraction to the wound nor for their activation. Together, these results suggest that extracellular ATP is not necessary for either macrophage recruitment or activation after the fin fold amputation.

### NF-κB Pathway Is Involved in Macrophage Activation After Wounding

**Figure 6:**
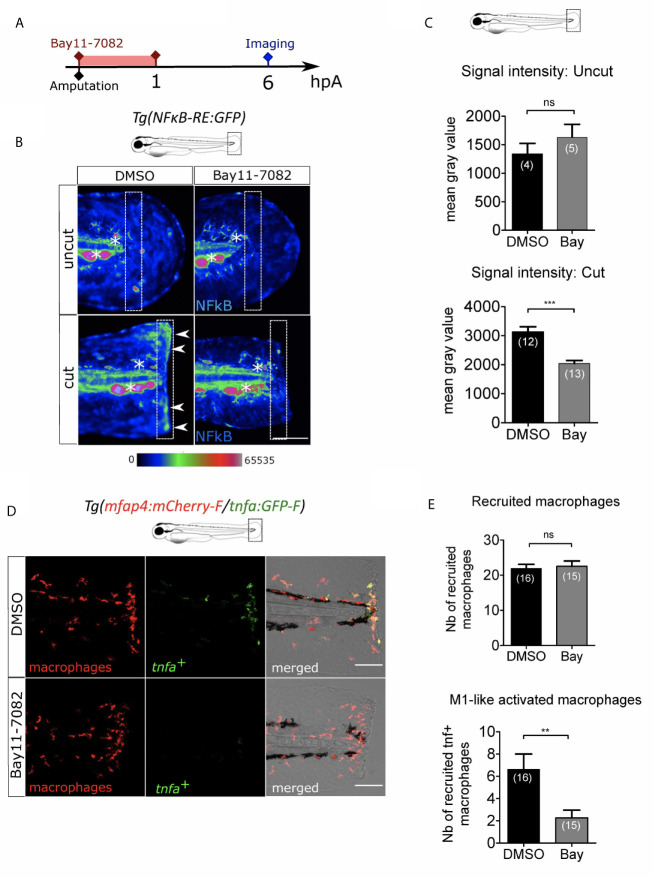
NFκB pathway is enrolled in macrophage activation after wounding. **(A)** Schedule of the experiment. Fin folds from *Tg(NFκB-RE : GFP)* or *Tg(mfap4:mCh-F/tnfa:GFP-F)* were amputated at 3 dpf and immediately treated either with DMSO or Bay11-7082 during 1 h. The drug was removed and larvae were imaged at 6 hpA using epi-fluorescent or confocal microscopy. **(B)** Representative images of the GFP fluorescence in uncut or cut fin folds from *Tg(NFκB-RE : GFP)* at 6 hpA, after the treatment with DMSO or Bay11-7082, detecting the NF-κB activation. Rainbow color scale was applied to images, emphasizing the differences in signal intensity. Asterisks show GFP signal in neuromast that is independent on NF-κB signaling. Arrow heads show NF-κB -dependent GFP signal at the wound edge. The white boxes outline the region of quantification. Scale bar: 100 μm. **(C)** Quantification of signal intensity of GFP fluorescence by mean gray value. Representative experiment of two independent experiments, mean ± SEM, *n_larvae_* is indicated in brackets, upper graph: Mann Whitney test, two-tailed, bottom graph: one-tailed t-test, ***p<0.001. **(D)** Tail images are representative maximum projections of the fluorescence of mCherry-F (macrophages), GFP-F (*tnfa^+^* cells) and merged channel images with brightfield of *Tg(mfap4:mCh-F/tnfa:GFP-F)* injured larvae after DMSO or Bay11-7082 treatment at 6 hpA. Scale bars: 100 μm. **(E)** Quantification of recruited macrophages (up) and *tnfa***^+^** recruited macrophages (middle) in controls and in DMSO or Bay11-7082 treated larvae at 6 hpA. Representative experiment of three independent experiments, mean ± SEM, *n_larvae_* is indicated in brackets, upper graph: two-tailed t-test, ns – not significant, bottom graph: two-tailed t-test with Welch’s correction, **p<0.01.

ROS are very small and easily diffusible signaling molecule that can pass through cell membranes to activate distant processes. Several redox-sensitive sensors have been proposed so far. NFκB is a major transcription factor that regulates key genes during inflammation. The signaling pathway leading to NFκB activation has been described to be redox sensitive (54) and was shown to be dependent on Ca²⁺-ROS axis at the wound (27). We thus hypothesized that NFκB signaling pathway would be a reasonable candidate to regulate macrophage activation. In many models including zebrafish, NFκB pathway can be specifically inhibited. For this, we used NF-κB inhibitor, Bay11-7082 (55). Firstly, we used the reporter line *Tg(NFkB-RE:eGFP)* to determine the dynamics of NF-κB activation. In normal condition some cells of the tail, like neuromast cells constitutively express GFP, as previously shown (56). We treated these larvae by adding Bay11-7082 directly in the medium during one hour immediately after the injury of the fin fold and measured the fluorescence of the reporter at 6 hpA (**Figure 6A**). In DMSO-treated larvae, the fluorescence intensity of cut fin fold was higher at the wound edge than in uncut condition, showing the activation of NF-κB pathway at the wound margin, as previously described. However, after Bay11-7082 treatment, the fluorescence intensity at the wound was lower compared to DMSO-treated wounded larvae (**Figures 6B, C**). These results confirmed the treatment efficacy in abolishing NF-κB activation at the wound. To assess the role of NF-κB on macrophage recruitment and activation, we treated *Tg(mfap4:mCherry-F/tnfa:GFP-F)* in the same way with Bay11-7082 (**Figure 6A**). We showed that Bay11-7082 had no effect on the number of macrophages recruited to the wound, but significantly decreased number of *tnfa* ^+^ macrophages (**Figures 6D-E**). Altogether, these data reveal that redox-sensitive NF-κB pathway is activated in response to tissue injury in zebrafish, and enrolled in macrophage activation at the injury site but not in recruitment.

### Macrophage-Expressed SFKs Lyn and Yrk Are Both Enrolled in Macrophage Activation

Besides NF-κB, Src family kinases (SFKs), a family of non-receptor tyrosine kinases, have been proposed to act as redox sensors through oxidation of their cysteine residues. Many SFKs are expressed in immune cells and play various roles in immunity (57). Their distinct role during macrophage activation at the wound is still elusive. To investigate the role of SFKs during macrophage activation in response to a wound, we used the general SFK inhibitor PP2. *Tg(mfap4:mCherry-F/tnfa:GFP-F)* larvae were injured and immediately treated with PP2 before being analyzed by microscopy for macrophage recruitment and activation at 6 hpA (**Figure S5A**). While PP2 treatment didn’t affect global macrophage population (**Figure S5B**), it caused strong reduction of macrophage recruitment at the wound, as previously shown (49). More importantly, PP2 treatment completely abolished macrophage activation, as indicated by *tnf*a^+^ macrophages at the wound (**Figures S5C-D**). This data confirmed that SFK activity is essential for the process of macrophage activation.

SFKs comprise many members whose function has been, for some of them, studied in zebrafish (49). To identify the SFK members important in ROS sensing by macrophages, we selected 5 SFKs: Lyn, Yes-related kinase (Yrk), Fyn, Hck and Yes. We analyzed published datasets of single-cell RNA sequencing data from adult zebrafish blood cells (58). In adult zebrafish, Lyn is expressed in both zebrafish macrophage and neutrophils with higher levels in macrophages while Yrk is expressed in both cell types in similar levels (**Figure S6**). In addition, Hck and Yes were also detected in adult macrophages. Published datasets of RNA sequencing performed on larval neutrophils and macrophages confirmed the expression of Lyn and Yrk in larval macrophages, while Hck and Yes mRNAs were not enriched in macrophages (59). We thus focused on Lyn and Yrk for further experiments.

**Figure 7:**
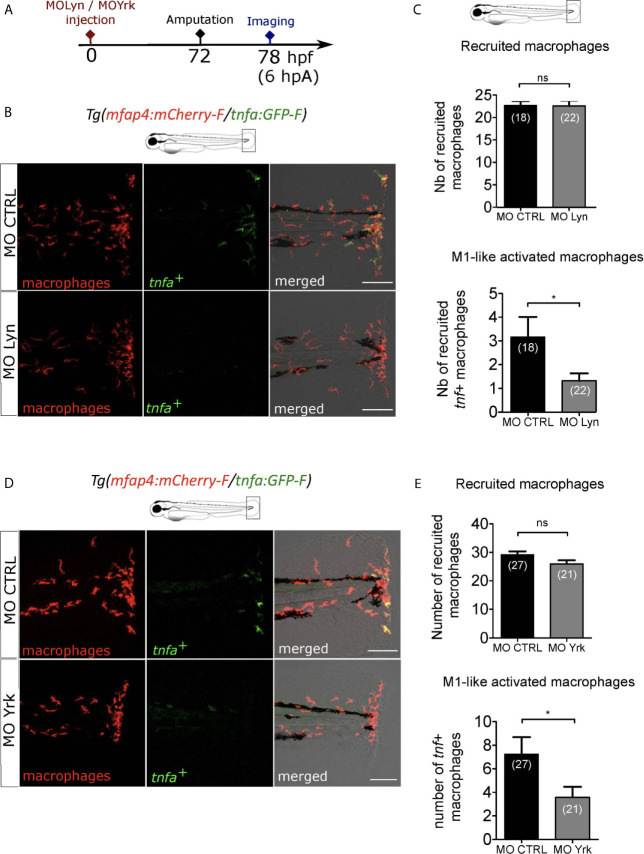
Macrophage-expressed SFKs Lyn and Yrk are enrolled in macrophage activation. **(A)** Schedule of the experiment. Morpholinos targeting specifically *lyn* (MO Lyn) or *yrk* (MO Yrk) or morpholino control (MO CTRL) were injected in *Tg(mfap4:mCherry/tnfa:GFP-F)* at one-cell stage. At 3 dpf, fin folds were amputated and larvae were imaged at 6 hpA using confocal microscopy. **(B)** Tail images are representative maximum projections of the fluorescence of mCherry-F (macrophages), GFP-F (*tnfa* ^+^ cells) and merged channel images with brightfield at 6 hpA, after the injection of MO CTRL (up) or MO Lyn (down). Scale bar: 100 μm. **(C)** Quantification of recruited macrophages (up) and *tnfa***^+^** recruited macrophages (down) in controls and in Lyn morphants at 6 hpA. Two independent experiments merged, mean ± SEM, *n_larvae_* is indicated in brackets, upper graph: two-tailed t-test, ns – not significant, bottom graph: Mann Whitney test, two-tailed, *p<0.05. **(D)** Tail images are representative maximum projections of the fluorescence of mCherry-F (macrophages), GFP-F (*tnfa*+ cells) and merged channel images with brightfield at 6 hpA, after the injection of MO CTRL (up) or MO Yrk (down). Scale bar: 100 μm. **(E)** Quantification of recruited macrophages (up) and *tnfa***^+^** recruited macrophages (down) in controls and in *yrk* morphants at 6 hpA. Two independent experiments merged, mean ± SEM, *n_larvae_* is indicated in brackets, two-tailed t-test, ns – not significant, *p<0.05.

While Lyn was previously shown to drive neutrophil attraction to the wound but not macrophage recruitment, Yrk was demonstrated to be required for macrophages recruitment (26, 49). To investigate the role of *lyn* in macrophage activation, we injected anti-sense oligonucleotides, morpholinos, which specifically block the splicing of *lyn* mRNA (MOLyn) in one-cell stage *Tg(mfap4:mCherry-F/tnfa:GFP-F)* embryos (**Figure 7A**). The efficiency of MOLyn was confirmed by RT-qPCR at 3 dpf (**Figure S7A**) and at this stage MOLyn did not induce any morphological defects (not shown). Subsequently, the fin fold of the morphants and their controls were injured and macrophages were analyzed by microscopy at 6 hpA (**Figure 7A**). As expected, Lyn morphants did not have any change in the number of recruited macrophages but had a significant decreased number of *tnfa* ^+^ macrophages at the wound, compared to control morphants (**Figures 7B, C**). To test the role of Yrk on macrophage function, we used a morpholino targeting the splicing of *yrk* mRNA (MOYrk). The efficiency of MOYrk was confirmed by RT-qPCR and sequencing at 3 dpf (**Figure S7B**) and the global morphology of the morphants was analysed. To test whether *yrk* knockdown affects macrophage recruitment as previously reported (49), we injected 2 doses of MO*yrk* morpholino in one-cell stage *Tg(mfap4:mCherry-F)* embryos (**Figure S7C**). In both 0.25 mM and 0.5 mM MO*yrk*, no morphological defects were observed (not shown). Subsequently, the fin fold of *yrk* morphants and their controls were injured at 3 dpf and imaged at 6 hpA. The number of recruited macrophages was decreased using 0.5 mM of MO*yrk* only, showing that the effect of *yrk* morpholino was dose-dependent (**Figure S7C**). This was not due to a global decrease of macrophages in *yrk* morphants as this number remained constant in the both 0.25 mM of 0.5 mM conditions compared to controls (**Figure S7D**). To test whether Yrk was also involved in macrophage polarization, we injected the lowest dose MO*yrk* (0.25mM) in one-cell stage *Tg(mfap4:mCherry-F/tnfa:GFP-F)* embryos and injured the fin fold as before at 3 dpf and imaged them at 6 hpA. Similarly to Lyn morphants, *yrk* morphants displayed a significant decreased number of *tnfa* ^+^ macrophages at the wound, compared to controls (**Figures 7D, E**).

Altogether these data show the mutual role of two different SFKs, Lyn and Yrk, in the process of pro-inflammatory macrophage activation after the tissue injury.

### Reactive Oxygen Species Interacts With Lyn But Not Yrk to Induce Macrophage Activation

The link between H_2_O_2_ and calcium signaling during wound healing has been debated previously. In *Drosophila* and zebrafish model, calcium plays an important role in the activation of Duox1 *in vivo* and H_2_O_2_ release (25, 27). However, in zebrafish, it has also been reported that H_2_O_2_ and calcium are two early signals that appear after tissue injury and are required for regeneration of the tissues, but act independently (28). To test whether calcium acts as upstream signal of ROS production in our system, larvae were injured at 3 dpf and stained with CellRox in the presence of Thapsigargin or DMSO. Imaging of wounded tails revealed that Thapsigargin did not affect CellRox signal at the wound edge (**Figures S8B, C**), suggesting that calcium transients are not required for ROS release, as previously shown in (28).

H_2_O_2_ has been proposed to activates the NF-κB inflammatory signaling pathway (27). To dissect the relationship between H_2_O_2_, calcium and NF-κB, *Tg(NFkB-RE : GFP)* larvae were wounded and immediately treated with either Apocynin, Thapsigargin or DMSO, then tails were imaged at 6 hpA. While Apocynin efficiently decreased NF-κB activity at the wound edge, compared to controls, Thapsigargin treatment did not change this NF-κB activity (**Figures S8D, E**), strongly suggesting that ROS but not calcium promotes the local activation of NF-κB.

**Figure 8:**
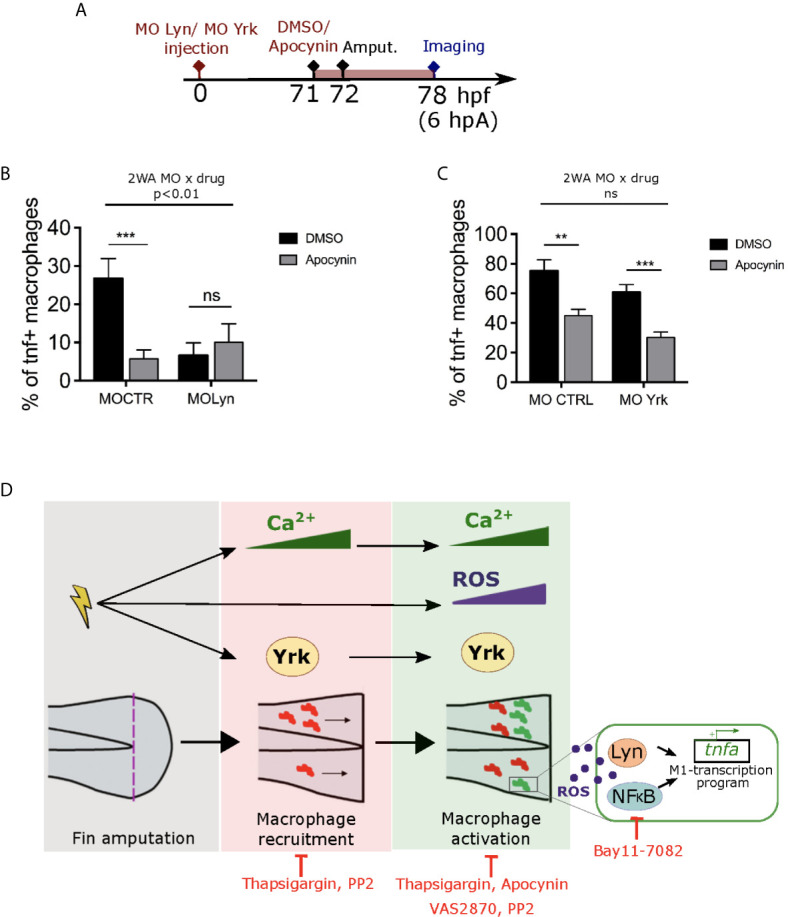
ROS interact with Lyn but not Yrk to mediate macrophage M1-like activation. **(A)** Schedule of the experiment. Morpholinos targeting specifically *lyn* (MO Lyn) or *yrk* (MO Yrk) or morpholino control (MO CTRL) were injected in *Tg(mfap4:mCherry/tnfa:GFP-F)* at one-cell stage. At 3 dpf (71 hpf), larvae were treated with Apocynin or DMSO, 1 hour before fin fold amputation. At 6 hpA, tails were imaged using confocal microscopy. **(B)** Quantification of the percentage of *tnfa^+^* macrophages in the recruited macrophage population at 6 hpA in CTRL morphants or *lyn* morphants which were either treated with DMSO or Apocynin (mean ± SEM, n_larvae_ is indicated in brackets). A significant interaction between morpholino and drug effect was determined by two-way ANOVA (2WA, **p<0. 01, F(1, 42) = 8.305). Then Mann Whitney test, two-tailed was performed to determine the significant difference between DMSO and Apocynin groups (ns-not significant, ***p<0.001). **(C)** Quantification of the percentage of tnfa^+^ macrophages in the recruited macrophage population at 6 hpA in CTRL morphants or *yrk* morphants which were treated with DMSO or Apocynin. (Representative experiment of two independent experiments, mean ± SEM, *n_larvae_* is indicated in brackets). No significant (ns) interaction between morpholino and drug effect was determined by two-way ANOVA (2WA, F(1,29)=0.0002613). Then Mann Whitney test, two-tailed was performed to determine the significant difference between DMSO and Apocynin groups (**p<0.01, ***p<0.001). **(D)** A proposed model showing the role of early wound signals in macrophage activation. Fin fold amputation triggers different independent stimuli: 1/intracellular Ca^2+^ oscillations in epithelial cells at the wound margin, which further mediate both macrophage recruitment and pro-inflammatory activation. 2/ROS production (mainly H_2_O_2_) at the wound, which promotes macrophage pro-inflammatory activation and *tnfa* expression. 3/Activation of SFK Yrk, which promotes both macrophage recruitment and activation in an independent manner. Redox-sensitive transcription factor NFκB and SFK Lyn are activated by ROS and required for M1-like activation at the wound. Thapsigargin and PP2 treatments impair both macrophage recruitment and M1-like activation. Apocynin, VAS2870 and Bay11-7082 affect only macrophage M1-like activation.

Previously, Lyn was shown to acts as a redox-sensor in neutrophils through oxidation (46). We have shown that the SFKs, Lyn and Yrk are important for the process of pro-inflammatory macrophage activation. However, whether these SFK are in the same signaling pathway than ROS or whether they act in parallel to trigger macrophage activation is still unknown. To test a putative crosstalk, we tested CTRL or Lyn morpholino injections with or without Apocynin inhibitor treatment on *Tg(mfap4:mCherry-F/tnfa:GFP-F)* larvae, that were then wounded and imaged 6 hours later (**Figure 8A**). Combination of drug and morpholino showed that Apocynin had a significant different effect on macrophage polarization depending on the morpholino. Indeed, the percentage of M1-activated macrophages was decreased in CTRL morphants, while it was unchanged in *Lyn* morphants (**Figure 8B**). Interestingly, a similar experiment was performed using Yrk morpholino. It shows that the effect of Apocynin on macrophage polarization was similar and did not depend on the injected morpholino. Indeed, the percentage of M1-activated macrophages was decreased in both CTRL and *yrk* morphants (**Figure 8C**).

Altogether, these results strongly suggest that ROS and calcium act independently and that ROS promotes macrophage M1-like activation though NF-κB and Lyn. Yrk, another SFK, is also involved in macrophage M1-like activation but it might act in parallel.

## Discussion

Based mainly on *in vitro* studies, many DAMPs have been proposed to drive immune cells to the wound and trigger their activation (22). In this study, we show that early signals induced by fin fold wounding in zebrafish control both macrophage recruitment and activation. The first identified signal is calcium. Indeed, preventing Ca²⁺ oscillations at the wound, using Thapsigargin treatment, impairs macrophage migration. This result is reminiscent of what happens during neuronal injury where rapidly propagating Ca^2+^ waves in the zebrafish brain determines which microglia will move to the damaged area (29). Intracytoplasmic calcium is thought to work as a second messenger leading to the activation of downstream molecules and several molecules have been proposed to induce and/or propagate calcium oscillation (60). Indeed, wound-induced calcium waves were shown to be dependent on extracellular ATP, through the binding to purinergic receptors, leading to the recruitment of neutrophils (27). Although macrophages can also respond rapidly to extracellular ATP *in vivo* and *in vitro* through the activation of P2Y receptors and P2X channels (61, 62), ATP does not seem to be required for macrophage recruitment nor activation in our system, as suppressing extracellular ATP at the wound using Apyrase treatment does not affect macrophage attraction to the wound, nor the expression of *tnfa*, while it does prevent neutrophil recruitment. The subject of future studies will be whether other nucleotides or amino acids are required in this process. Further, calcium signaling has been studied in the context of macrophage function (61) and, besides chemotaxis, Ca^2+^ was shown to regulate other aspects of macrophage function like phagocytosis (63, 64). Our data show that during wound healing calcium signaling is also required for proper M1-like polarization at the injury site, as preventing calcium oscillations at the wound also prevents the expression of *tnfa* and *il1b* in macrophages. Although Ca^2+^ waves appear and spread in the epithelial cells of the wound (25, 28, 50), Ca^2+^ oscillations can also occur in other cell types. Recently, a study of calcium dynamics within neutrophils showed that wound-induced inflammation triggers different calcium signals in migrating versus clustered neutrophils. The authors proposed that the “calcium alarm” signal in neutrophil clusters contributes to effectively close damaged tissue barriers to stop pathogen entry (65). However, neutrophils are not involved in the regulation of macrophage M1-like activation (see below), therefore we propose that they are not the source of calcium signals in this context. Recently, in zebrafish infection model, it was shown that *Mycobacterium marinum* increases Ca^2+^ oscillations in macrophages through purinergic receptor P2RX7 (66). Whether calcium oscillations also occur in macrophages of the wound requires further investigations.

Other early wound signals are the reactive oxygen species (ROS), mainly H_2_O_2_, which are produced at the wound within minutes after an injury (24, 46). By manipulating ROS concentrations using inhibitors of NADPH oxidases or H_2_O_2_ supplementation in the medium, we demonstrated that wound induced-ROS are required for macrophage activation, as inhibition of NADPH oxidases significantly decrease *tnfa* production in macrophages. In the other hand, the increase of H_2_O_2_ concentration in the tissues impairs both macrophage recruitment and *tnfa* expression in macrophages, suggesting narrow windows of H_2_O_2_ concentrations are key to macrophage function. ROS promote wound healing and regeneration in different models and manipulation of H_2_O_2_ concentrations impacts the fate of the wound. Indeed, attenuation of H_2_O_2_ thanks to catalase overexpression leads to the delay of wound healing in mice (67) and inhibition of ROS production using pharmacological agents blocks regeneration in zebrafish (28, 68, 69). By contrast, low levels of H_2_O_2_ are known to promote cutaneous sensory axon repair in injured zebrafish (53) and to accelerates wound closure and angiogenesis in mice (70). Our data add to the increasingly diverse roles of ROS in the context of wounds and highlights the importance of these oxidizing molecules on wound-macrophage plasticity.

The main source of ROS at the wound is the NADPH oxidase Duox present in epithelial cells (24). In zebrafish NOX2 is also present in leukocytes, and was shown to be involved in macrophage recruitment to the wound (49) but not neutrophils (24). However, our present observations diverge from previous studies (49) as knocking down *p47^phox^*, a NOX2 subunit, does not abolish macrophage recruitment nor activation, suggesting that *p47^phox^* is not involved in these processes. We therefore propose that Duox is the NADPH oxidase required for macrophage activation. Although H_2_O_2_ was proposed to act downstream of calcium signaling (27), we showed here that calcium signal inhibition did not impair ROS production, suggesting that calcium and ROS act independently (**Figure 8D**). This is reminiscent to a previous study in zebrafish where Thapsigargin treatment did not block H_2_O_2_ burst at the wound (28) Accumulating evidences speak for a role of ROS in regulating the functional polarization of macrophages. Indeed, a reduced Nox2 promotes M2 polarization and the mutation in *p47^phox^* in mice favors the macrophage reprogramming towards M2 phenotype (71). Furthermore, TLR activation was shown to prime the generation of mitochondrial ROS production which favors M1-polarization (72). ROS production depends on immunoresponsive gene 1 (*irg-1*) whose depletion impairs the hallmarks of M1 macrophages: fatty acid mobilization and bactericidal activity (73). Although the source of ROS is different, all these studies emphasize the important role of ROS in macrophage polarization.

How ROS drive macrophage polarization *in vivo* is still poorly understood. Due to its stability and ability to easily pass through cellular membrane, the ROS, H_2_O_2_ has been proposed to be an important signaling molecule that can regulate downstream signaling pathways through oxidation of proteins. In our study, we studied 3 redox-sensitive proteins present in macrophage that can potentially mediate ROS regulation: NF-κB, Lyn and Yrk. NF-κB was previously shown to be a redox-sensitive transcription factor, regulated by ROS through IκB-kinase activation and IκBα tyrosine phosphorylation (54) and, in zebrafish, H_2_O_2_ was shown to activate NFκB pathway in injured fin folds (27). In this study, we show that inhibition of ROS production with Apocynin, abolishes NF-κB signal at the wound, while inhibition of calcium with Thapsigargin has no effect. In addition, using the NF-κB specific inhibitor Bay11-7082, we show that blocking NF-κB pathway leads to a decrease of macrophage M1-like activation without affecting their migration. Yet, two scenarios are possible to explain NF-κB mediated M1-like identity. In zebrafish larva, NF-κB activation has been reported in epithelial cells at the wound edge (56, 74) and in macrophages (75). However, NF-κB is the major transcription factor controlling tnfa and il1b expression and is one of the defining transcription factors of an M1-like identity in macrophages in other systems. We thus speculate that wound-induced ROS activate NF-κB in macrophages, leading to the upregulation of pro-inflammatory gene expression corresponding to M1-polarization (**Figure 8D**).

SFKs are protein tyrosine kinases involved in multiple signaling pathways where they play critical roles in the regulation of leukocyte function (76). The Lyn and Yrk SRC family kinases (SFKs) are both expressed in zebrafish macrophages (49) and database from (59) and (58). In this study, while general inhibition of SFKs with PP2, impairs both macrophage migration and polarization toward M1-like phenotypes, the specific knock down of *lyn* using morpholino, only affects macrophage activation. The role of Lyn has been intensively studied in murine immune cells including macrophages, where it regulates myeloproliferation and sensitivity to growth factors. In combination with Hck, Lyn also controls M2 macrophage polarization (77). Upon inflammation, Lyn was proposed to act as an inflammation-sensitive checkpoint determining activation in mouse bone marrow-derived macrophages (78). Our data are in accordance with these results. Whether Lyn mediates polarization directly acting in macrophages or in other cell types remains to be determined. By contrast, the role of Yrk in macrophages is poorly known and its expression is hardly detected in mouse macrophages (76). In zebrafish, Yrk morpholino impairs macrophage recruitment [(49) and present study]. However, the effect of the morpholino on macrophage migration was dose-dependent suggesting that the level of expression of Yrk needs to be tightly regulated during wound healing. In addition, Yrk morpholino affects macrophage polarization at the wound showing a new function for Yrk in immune regulation. Recently, SFKs, and more specifically Lyn in neutrophils, were shown to act as redox sensor that detects H_2_O_2_ (46, 79, 80). By using simultaneously morpholino injections and ROS inhibitors, we found that Lyn acts in the same pathway as ROS to trigger macrophage M1-like polarization at the wound. By contrast Yrk, whose knockdown has an additive effect to Apocynin on M1-like activation, may not be involved in the same pathway as ROS. Yrk may work also independently of calcium signaling as a previous report showed that in the tail wounding model, Thapsigargin has no effect on SFK activation (28) (**Figure 8D**).

While macrophage were shown to be important for neutrophil function (49, 81), neutrophils seem to be dispensable for macrophage activation. This is supported by genetic depletion of neutrophils which did not alter macrophage activation nor recruitment, but also with apyrase treatment, where neutrophil infiltration is impaired while macrophage accumulation and polarization state appear normal.

Calcium and H_2_O_2_ are widely-conserved early wound signals, present not only in zebrafish (24, 27, 28) but also flies (25) and mammals (82). A recent study in Drosophila revealed that of H_2_O_2_ produced from a wound lead to the activation of hemocytes, which are the equivalent of macrophages in fly. This activation is characterized by the activation of Toll signaling and the induction of the cytokine-like protein upd3, through the action of the Tyrosin-protein Kinase Src42A, the Drosophila Lyn homolog (83). We propose that early wound signals may play a conserved role in macrophage polarization in arthropods and vertebrates during wound healing, as ancient triggers of inflammation. Dissecting those pathways will provide insights into how we might modulate macrophage responses during pathological wounds or trauma.

## Data Availability Statement

All numerical data created during this study are openly available from Zenodo repository at http://doi.org/10.5281/zenodo.4581166.

## Ethics Statement

The animal study was reviewed and approved by Comité d’Ethique pour l’Expérimentation Animale under reference CEEA-LR- B4-172-37 and APAFIS#5737-2016061511212601 v3.

## Author Contributions

TS performed conception and design, acquisition of data, analysis, interpretation of data and co-wrote the manuscript. RH-A performed conception and design, acquisition of data, analysis and interpretation of data. RP and MG performed acquisition of data and analysis. CG, CB-P and FE performed acquisition of data, analysis and provided laboratory animals and samples. GL contributed to conception and design and revised the manuscript critically for important intellectual content. MN-C performed conception and design, interpretation of data and co-wrote the manuscript. All authors contributed to the article and approved the submitted version.

## Funding

This work was supported by a grant from the European Community’s H2020 Program [Marie-Curie Innovative Training Network ImageInLife: Grant Agreement n° 721537], by a grant from the french Agence Nationale de la Recherche [ANR-19-CE15-0005-01, MacrophageDynamics] and by a grant from REPERE ImageInLife de la region Occitanie. Funding sources had no role in the writing of the manuscript or the decision to submit it for publication.

## Conflict of Interest

The authors declare that the research was conducted in the absence of any commercial or financial relationships that could be construed as a potential conflict of interest.
