# Supplementary material for: Damage-Induced Calcium Signaling and Reactive Oxygen Species Mediate Macrophage Activation in Zebrafish
Source: Front Immunol. 2021 Mar 26;12:636585. doi: 10.3389/fimmu.2021.636585 (PMC8032883; doi:10.3389/fimmu.2021.636585)
Supplement: Supplementary file 1 [file DataSheet_1.pdf]

Supplementary Data

Damage-induced Calcium signalling and Reactive Oxygen Species mediate macrophage activation in zebrafish

Tamara Sipka[1], Romain Peroceschi[1$], Rahma Hassan-Abdi[1$], Martin Groß[1], Felix Ellett[2,3], Christina Begon-Pescia[1], Catherine Gonzalez[1], Georges Lutfalla[1], Mai Nguyen-Chi[1] *

[1]LPHI, Univ Montpellier, CNRS, Montpellier, France

[2]Bateson Centre and Department of Infection and Immunity, University of Sheffield, Sheffield, United Kingdom

[3]BioMEMS Resource Center, Center for Engineering in Medicine and Surgery, Massachusetts General Hospital and Harvard Medical School, Boston, Massachusetts 02114, USA

[$]These authors contributed equally to this work

*** Correspondence:**
Mai Nguyen-Chi
mai-eva.nguyen-chi@umontpellier.fr

SUPPLEMENTARY FIGURE LEGENDS:

Figure S1: **Thapsigargin treatment impairs oscillations of intracellular Ca^{2+} concentration and decrease macrophage recruitment/activation at the wound**

(A) Representative images of the Fluo-3-AM fluorescence in uncut or cut fin folds, immediately after the fin fold amputation, showing Ca^{2+} production at the wound margin. Rainbow color scale was applied to images, emphasizing the differences in signal intensity (N=10 larvae per group, two independent experiments). The white lines outline the fin fold and the notochord. Scale bar: 50 µm.

(B) Schedule of the experiment. At one-cell stage, larvae were injected with pGFP_CMV:GCaMP6s plasmid. At 3 dpf, positive larvae were selected and treated with Thapsigargin or DMSO for 6 h, then injured and imaged from 5 to 15 min post injury every 20 seconds, using Spinning-disk confocal microscopy.

(C) Visualisation of intracytoplasmic calcium oscillations using GCaMP6s sensor (green) in epithelial cells of the fin fold after injury. Representative frames are maximum projections of GCaMP6s fluorescence from a video in larvae that were either treated with DMSO or with Thapsigargin, showing oscillations of intracellular Ca^{2+} concentration. Time step: 20 s. Scale bars: 50 µm.

(D) Quantification of fluorescence intensity (mean gray value) of two GCaMP6-expressing cells in larvae treated with DMSO (up) or Thapsigargin (down), in each time-step (20 seconds), showing the oscillations in intracellular Ca^{2+} concentration after the injury over the time.

(E) Quantification of total macrophages in uninjured *Tg(mfap4:mCherry/tnfa:GFP-F)* larvae at 3 dpf after DMSO or Thapsigargin treatments. Thapsigargin does not influence the total number of macrophages. Mean ± SEM, n_{larvae} is indicated in brackets, two-tailed t-test, ns – not significant.

Figure S2. ROS release at the wound mediates macrophage activation but not recruitment

(A) Schedule of the experiment**.** Larvae were injected in the yolk with VAS2870 or DMSO at 3 dpf, and 20 min after incubated in CellROX solution. After 1 h, fin folds were amputated. CellROX staining was removed at 20 min pA, and larvae were immediately imaged using epi-fluorescence microscopy.

(B) Representative images of the CellROX fluorescence in uncut or cut fin folds, after the treatment with DMSO or VAS2870 at 20 min pA, showing ROS production at the wound. Rainbow color scale was applied to images, emphasizing the differences in signal intensity. The white lines outline the fin fold and the notochord. Scale bar: 100 µm.

(C) Quantification of signal intensity the CellROX fluorescence by mean gray value. Representative experiment of three independent experiments, mean ± SEM, n_{larvae} is indicated in brackets, upper

graph: Mann Whitney test, two-tailed, ns – not significant, bottom graph: two-tailed t-test, ***p<0.001.

(D) Schedule of the experiment**.** *Tg(mfap4:mCherry-F/tnfa:GFP-F)* larvae were injected in the yolk with VAS2870 or DMSO at 3 dpf. Fin folds were amputated 20 min later and imaged at 6 hpA using confocal microscopy.

(E) Tail images are representative maximum projections of the fluorescence of mCherry-F (macrophages), GFP-F (*tnfa*$^{+}$ cells) and merged channel images with brightfield in *Tg(mfap4:mCh/tnfa:GFP-F)* injured larvae after DMSO or VAS2870 treatment at 6 hpA. Scale bar: 100 µm.

(F) Quantification of recruited macrophages (up) and tnfa^{+} recruited macrophages (middle) in in DMSO or VAS2870 treated larvae at 6 hpA. Representative experiment of two independent experiments, mean ± SEM, n_{larvae} is indicated in brackets, upper graph: two-tailed t-test, ns – not significant, bottom graph: Mann Whitney test, two-tailed, **p<0.01.

Figure S3: Elevation of ROS concentration impairs macrophage recruitment to the wound and M1-like activation.

(A) Schedule of the experiment. Larvae were incubated in a CellROX (10µM) and H_2O_2 (2mM) solution or only a CellROX (10µM) solution (Control). After 1 h, fin folds were amputated. CellROX solutions were removed at 20 min pA, and larvae were immediately imaged using epi-fluorescence microscopy.

(B) Representative images of the CellROX fluorescence in uncut or cut fin folds, after the treatment without or with H_2O_2 at 20 min pA, showing ROS production at the wound. Rainbow color scale was applied to images, emphasizing the differences in signal intensity. The white lines outline the fin fold and the notochord. The yellow arrowheads show the intensity increase at the fin fold or wound area. Scale bar: 100 µm.

(C) Quantification of signal intensity of the CellROX fluorescence by mean gray value. Representative experiment of three independent experiments, mean ± SEM, n_{larvae} is indicated in brackets, Mann Whitney test, two-tailed, **p<0.01, ***p<0.001.

(D) Schedule of the experiment. Immediately after the fin fold amputation at 3 dpf, *Tg(mfap4:mCherry-F/tnfa:GFP-F)* larvae were incubated in H_2O_2 (2mM) or fish water for 6 h. Larvae were imaged at 6 hours post amputation (hpA) using confocal microscopy.

(E) Tail images are representative maximum projections of the fluorescence of mCherry-F (macrophages), GFP-F (*tnfa*$^{+}$ cells) and merged channel images of *Tg(mfap4:mCherry-F/tnfa:GFP-F)* injured larvae, after the incubation in H_2O_2 (down) or fish water (CTRL, up) at 6 hpA. Scale bars: 100 µm.

(F) Quantification of total macrophages in whole larvae at 3 dpf in indicated conditions. H_2O_2 treatment does not influence total number of macrophages. Mean ± SEM, n_{larvae} is indicated in brackets, two-tailed t-test, ns – not significant.

(G) Quantification of recruited macrophages (up) and percentage of *tnfa*$^+$ macrophages in the recruited population (down) after incubation in H_2O_2 or fish water (Control) at 6 hpA. Representative experiment of three independent experiments, mean ± SEM, n_{larvae} is indicated in brackets, Mann Whitney test, two-tailed, ***p<0.001.

Figure S4: Ros produced by Nox2 are not required for macrophage activation at the wound

(A) Schedule of the experiment**.** Morpholino targeting specifically p47^{phox} (MO p47^{phox}) or a morpholino control (MO CTRL) were injected in *Tg(mpx:GFP)* at one-cell stage. At 48 hpf, larvae were infected with *Escherichia coli* expressing GFP in the muscle and stained at 2 hpi with DHE before confocal imaging.

(B) Trunk images are representative maximum projections of merged channel images of DHE and GFP fluorescence (neutrophils) in control morphants (MO CTRL) or p47^{phox} morphants (MO p47^{phox}) at 2 hpi. Scale bar: 20 µm.

(C) Quantification of signal intensity of the DHE fluorescence by mean gray value. Representative experiment of two independent experiments, mean ± SEM, n_{larvae} is indicated in brackets, Mann Whitney test, two-tailed, *p<0.05.

(D) Schedule of the experiment**.** Morpholino targeting specifically p47^{phox} (MO p47^{phox}) or a morpholino control (MO CTRL) were injected in *Tg(mfap4:mCh-F/tnfa:GFP-F)* at one-cell stage. At 3 dpf, fin folds were injured and larvae were imaged at 6 hpA using confocal microscopy.

(E) Tail images are representative maximum projections of the fluorescence of mCherry-F (macrophages), GFP-F *(tnfa*$^+$ cells) and merged channel images with brightfield after the injection of MO CTRL (up) or MO p47^{phox} (down) at 6 hpA. Scale bar: 100 µm.

(F) Quantification of recruited macrophages (up) and tnfa$^+$ recruited macrophages (down) in controls and in p47^{phox} morphants at 6 hpA. Two independent experiments merged, mean ± SEM, n_{larvae} is indicated in brackets, upper graph: two-tailed t-test, bottom graph: two-tailed t-test with Welch's correction, ns - not significant.

Figure S5: SFKs are enrolled in macrophage recruitment and essential for M1-like activation

(A) Schedule of the experiment**.** Fin folds from *Tg(mfap4:mCh-F/tnfa:GFP-F)* were amputated at 3 dpf and larvae were immediately incubated in PP2 or DMSO solution until imaging at 6 hpA.

(B) Quantification of total macrophages in whole larvae at 3 dpf in indicated conditions. PP2 treatment does not influence total number of macrophages. Mean ± SEM, n_{larvae} is indicated in brackets, two-tailed t-test, ns – not significant.

(C) Tail images are representative maximum projections of the fluorescence of mCherry-F (macrophages), GFP-F (*tnfa*$^+$ cells) and merged channel images with brightfield of *Tg(mfap4:mCh-F/tnfa:GFP-F)* injured larvae after the treatment with DMSO (up) or PP2 (down) at 6 hpA. Scale bar: 100 µm.

(D) Quantification of recruited macrophages (up) and *tnfa*$^+$ recruited macrophages (middle) in DMSO or PP2 treated larvae at 6 hpA. Representative experiment of two independent experiments, mean ± SEM, n_{larvae} is indicated in brackets, upper graph: Mann Whitney test, two-tailed, **p<0.01.

Figure S6: Expression profiles of zebrafish SFKs in blood cells

Expression profile of neutrophil-specific *mpx* and macrophage-specific *mfap4* gene in adult blood cells extracted from (https://www.sanger.ac.uk/science/tools/basicz/basicz/) is presented on the left side of the panel. Expression profiles of selected zebrafish SFKs: Lyn, Yrk, Hck, Fyn and Yes are presented on right side, confirming that Lyn and Yrk are both expressed in macrophages.

Figure S7: Verification of MOLyn and MOYrk efficiency

(A) Analysis of *lyn* mRNA levels by RT–PCR from whole larvae extracts (pool of 15 larvae). Larvae were injected with Lyn morpholino (MO Lyn), anti-sense oligonucleotides targeting the Exon9-Intron9/10 site, or control morpholino (MO CTRL) at one-cell stage and Lysed for RNA extraction at 3 dpf. *ef1a* mRNA level was used as a control on same samples.

(B) Analysis of *yrk* mRNA levels by RT–PCR from whole larvae extracts (pool of 15 larvae). Larvae were injected with Yrk morpholino (MO Yrk), anti-sense oligonucleotides targeting Exon6-Intron6/7 site or control morpholino (MO CTRL) at one-cell stage and Lysed for RNA extraction at 3 dpf. *ef1a* mRNA level was used as a control on same samples.

(C) Tg(mfap4:mCherryF) embryos were injected with two different concentrations of Yrk morpholino (MO Yrk), 0.25 mM and 0.5 mM or with control morpholino (MO CTRL) at one-cell stage. At 3 dpf larvae were amputated and their tails were imaged at 6 hpA using fluorescent microscopy. The number of recruited macrophages was then quantified in the different conditions (mean ± SEM, n_{larvae} is indicated in brackets, Kruskal–Wallis test, *p<0.05).

(D) Quantification of total macrophages in truck and tail regions in indicated conditions. (mean ± SEM, n_{larvae} is indicated in brackets, Kruskal–Wallis test, *p<0.05).

Figure S8: Calcium transient inhibition does not impair ROS production nor NF-κB activity at the wound.

(A) Schedule of the experiment. 1h before the fin fold amputation at 3 dpf, larvae were incubated in Thapsigargin or DMSO, containing CellROX solution for the detection of the ROS production. Drug and

staining were both removed at 20 min pA, and larvae were immediately imaged using epi-fluorescent microscopy.

(B) Representative images of the CellROX fluorescence in uncut or cut fin folds, after the treatment with DMSO or Thapsigargin at 20 min pA. Rainbow color scale was applied to images, emphasizing the differences in signal intensity. The white lines outline the fin fold and the notochord. Scale bar: 100 µm.

(C) Quantification of signal intensity of the CellROX fluorescence by mean grey value. Representative experiment of two independent experiments, mean ± SEM, n_{larvae} is indicated in brackets, two-tailed t-test, ns – not significant.

(D) Schedule of the experiment**.** *Tg(NFκB-RE:GFP)* at 3 dpf was treated either with Apocynin 1h before the fin fold amputation until 6 hpA, or with Thapsigargin immediately after the amputation during 1 h. Larvae were imaged at 6 hpA using epi-fluorescent microscopy.

(E) Representative images of the GFP fluorescence in cut fin folds of *Tg(NFκB-RE:GFP)* at 6 hpA, after the treatment with DMSO, Apocynin or Thapsigargin, detecting the NF-κB activation. Rainbow color scale was applied to images, emphasizing the differences in signal intensity. Scale bar: 100 µm.

(F) Quantification of signal intensity of GFP fluorescence of *Tg(NFκB-RE:GFP)* line by mean grey value. Representative experiment of two independent experiments, mean ± SEM, n_{larvae} is indicated in brackets, two tailed t-test, ns – not significant, ***p<0.001.

MOVIE LEGENDS:

Movie 1: *Tg(mfap4:mCherry-F/tnfa:GFP-F)* line reporter allows imaging of macrophage M1-like activation at the wound in response to fin fold injury

Macrophage movements and activation states were imaged by confocal microscopy from 2 to 9.5 hours post amputation (hpA) using the Tg(mfap4:mCherry-F/tnfa:GFP-F) line. Video is composed of maximum projections of the overlaid fluorescences of mCherry-F (macrophages) and GFP-F ($tnfa^{+}$ cells), recorded every 3 minutes and showing M1-like activation of recruited macrophages at the wound. White line indicates the wound margin. Scale bar: 100 µm.

Movie 2: calcium oscillations after a wound

Visualization of intracytoplasmic calcium oscillations using GCaMP6s sensor (green) in epithelial cells of the fin fold after injury, as maximum projections of time frames recorded

every 20 seconds. Larvae were either treated with DMSO (up) or with Thapsigargin (down), showing oscillations of intracellular Ca^{2+} concentration. Cells selected for signal quantification are indicated with arrowheads. Scale bars: 50 µm.

Fig.S1

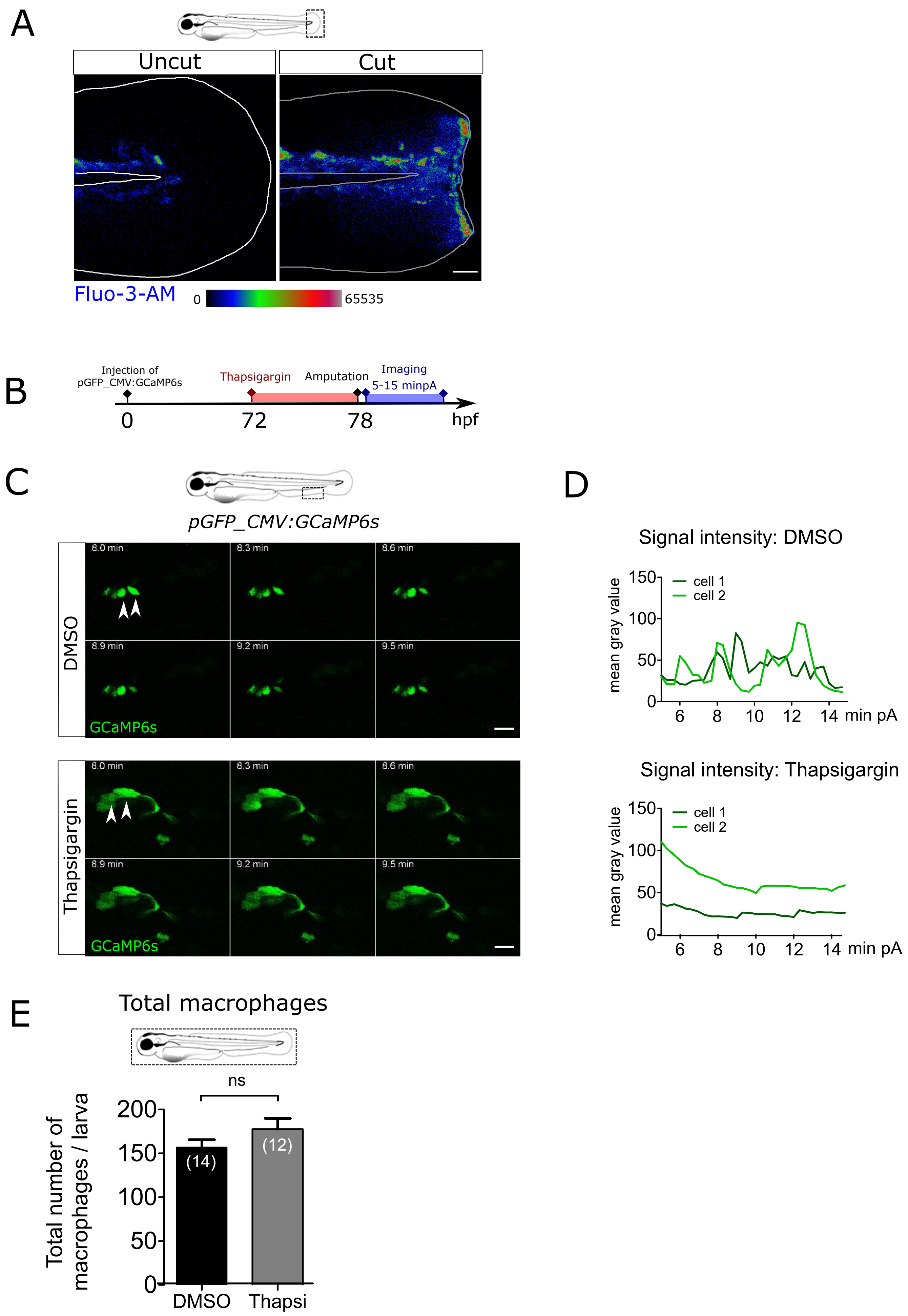

A
Uncut
Cut
Fluo-3-AM
0
65535
B
Injection of pGFP_CMV:GCaMP6s
Thapsigargin
Amputation
Imaging 5-15 minpA
0
72
78
hpf
C
pGFP_CMV:GCaMP6s
DMSO
Thapsigargin
8.0 min
8.3 min
8.6 min
8.9 min
9.2 min
9.5 min
GCaMP6s
D
Signal intensity: DMSO
Signal intensity: Thapsigargin
mean gray value
cell 1
cell 2
min pA
E
Total macrophages
ns
Total number of macrophages / larva
(14)
(12)
DMSO
Thapsi

Fig. S2

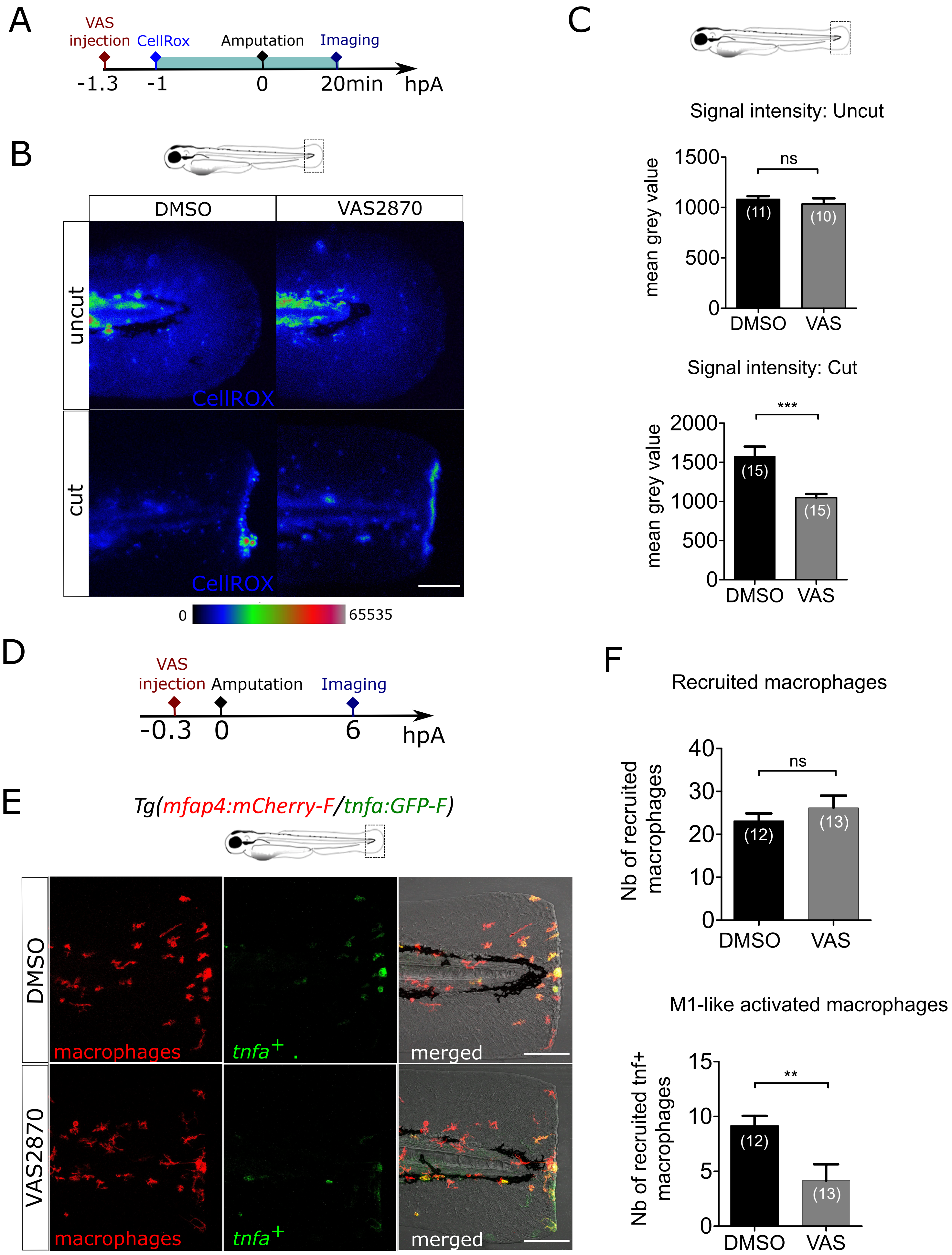

A
VAS injection
CellRox
Amputation
Imaging
-1.3
-1
0
20min
hpA
B
DMSO
VAS2870
uncut
cut
CellROX
CellROX
0
65535
C
Signal intensity: Uncut
mean grey value
1500
1000
500
0
ns
(11)
(10)
DMSO
VAS
Signal intensity: Cut
mean grey value
2000
1500
1000
500
0
***
(15)
(15)
DMSO
VAS
D
VAS injection
Amputation
Imaging
-0.3
0
6
hpA
E
Tg(mfap4:mCherry-F/tnfa:GFP-F)
DMSO
VAS2870
macrophages
tnfa+
merged
macrophages
tnfa+
merged
F
Recruited macrophages
Nb of recruited macrophages
40
30
20
10
0
ns
(12)
(13)
DMSO
VAS
M1-like activated macrophages
Nb of recruited tnf+ macrophages
15
10
5
0
**
(12)
(13)
DMSO
VAS

Fig S3

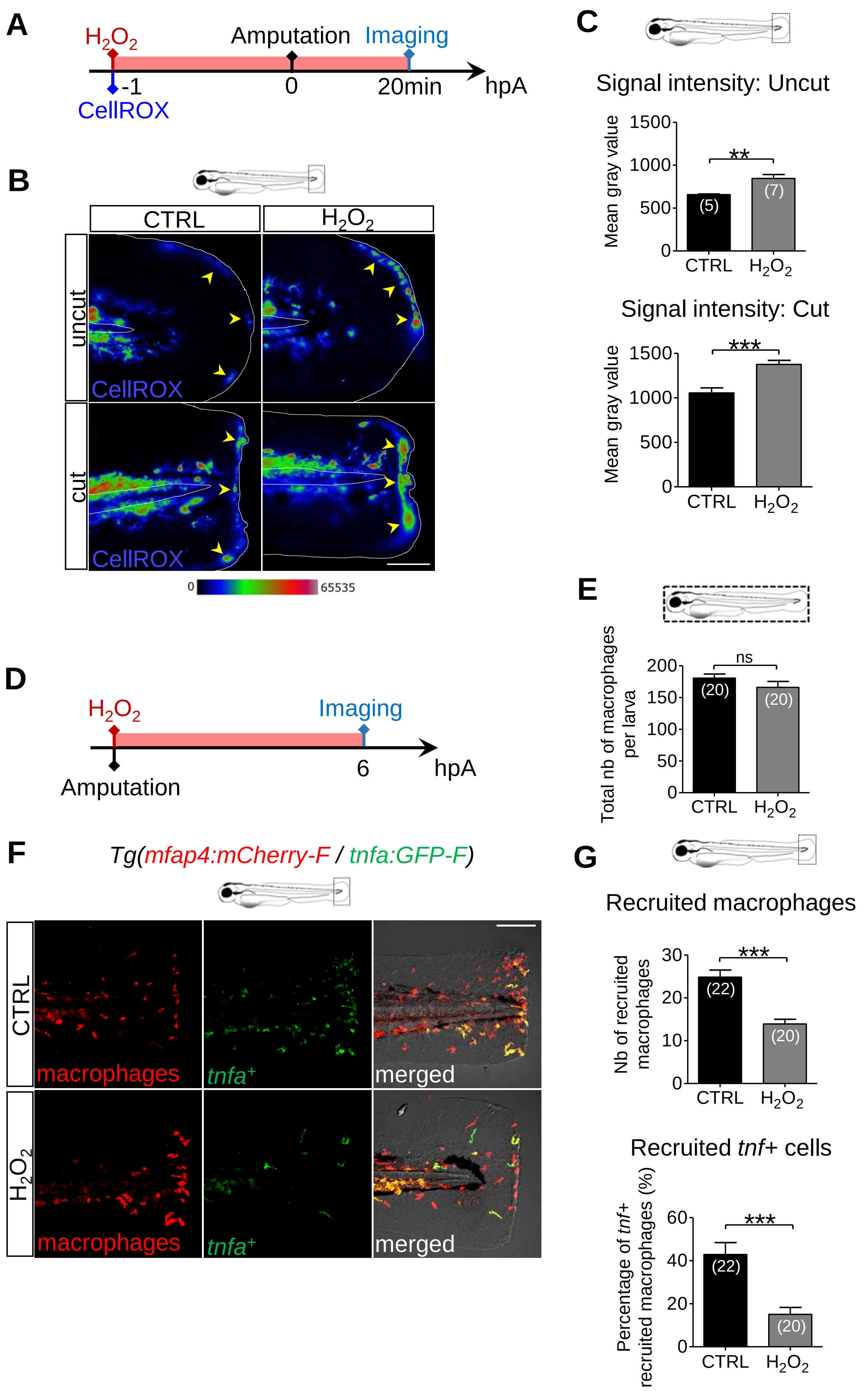
A
H_2O_2
Amputation
Imaging
-1
0
20min
hpA
CellROX
B
CTRL
H_2O_2
uncut
cut
CellROX
CellROX
0
65535
C
Signal intensity: Uncut
Mean gray value
1500
1000
500
0
**
(5)
(7)
CTRL
H_2O_2
Signal intensity: Cut
Mean gray value
1500
1000
500
0
***
CTRL
H_2O_2
D
H_2O_2
Imaging
Amputation
6
hpA
E
Total nb of macrophages per larva
200
150
100
50
0
ns
(20)
(20)
CTRL
H_2O_2
F
Tg(mfap4:mCherry-F / tnfa:GFP-F)
CTRL
H_2O_2
macrophages
$tnfa^+$
merged
macrophages
$tnfa^+$
merged
G
Recruited macrophages
Nb of recruited macrophages
30
20
10
0
***
(22)
(20)
CTRL
H_2O_2
Recruited tnf+ cells
Percentage of tnf+ recruited macrophages (%)
60
40
20
0
***
(22)
(20)
CTRL
H_2O_2

Fig. S4

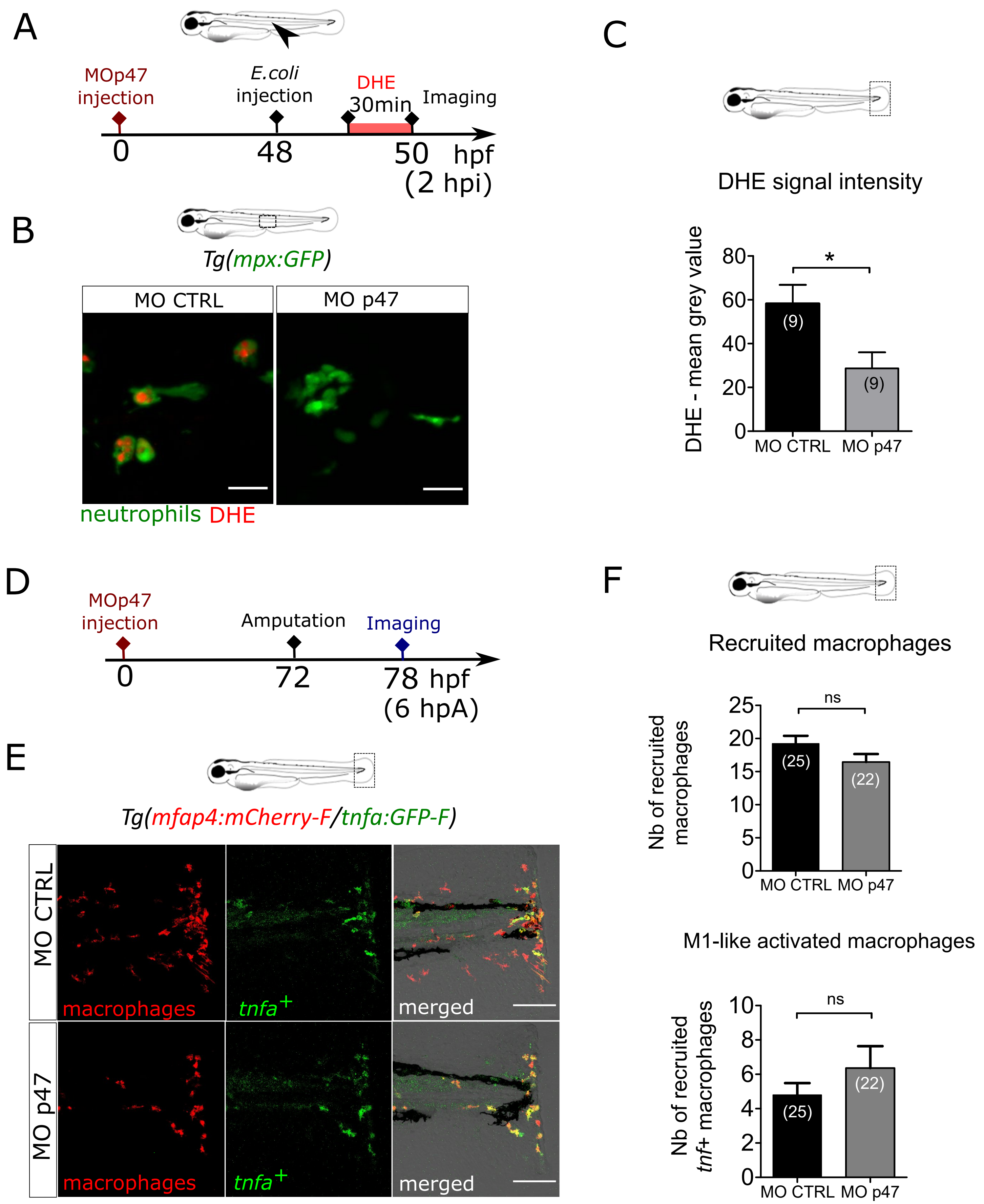

A
MOp47
injection
E.coli
injection
DHE
30min
Imaging
0
48
50
hpf
(2 hpi)
B
Tg(mpx:GFP)
MO CTRL
MO p47
neutrophils DHE
C
DHE signal intensity
DHE - mean grey value
80
60
40
20
0
*
(9)
(9)
MO CTRL
MO p47
D
MOp47
injection
Amputation
Imaging
0
72
78 hpf
(6 hpA)
E
Tg(mfap4:mCherry-F/tnfa:GFP-F)
MO CTRL
MO p47
macrophages
tnfa+
merged
F
Recruited macrophages
Nb of recruited macrophages
25
20
15
10
5
0
ns
(25)
(22)
MO CTRL
MO p47
M1-like activated macrophages
Nb of recruited tnf+ macrophages
10
8
6
4
2
0
ns
(25)
(22)
MO CTRL
MO p47

Fig. S5

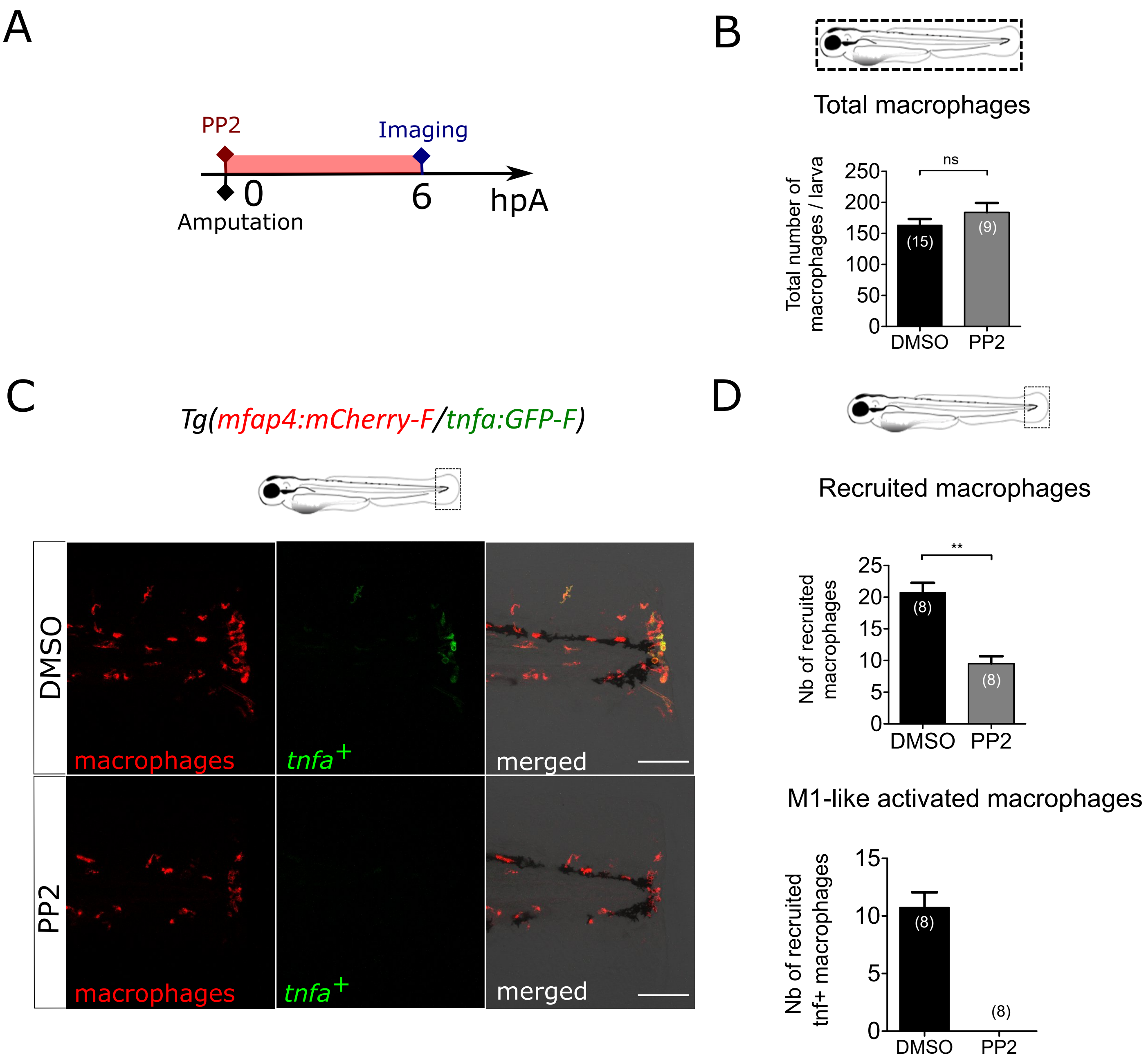
A
PP2
Imaging
0
6
hpA
Amputation
B
Total macrophages
Total number of macrophages / larva
250
200
150
100
50
0
ns
(15)
(9)
DMSO
PP2
C
Tg(mfap4:mCherry-F/tnfa:GFP-F)
DMSO
PP2
macrophages
tnfa+
merged
macrophages
tnfa+
merged
D
Recruited macrophages
Nb of recruited macrophages
25
20
15
10
5
0
**
(8)
(8)
DMSO
PP2
M1-like activated macrophages
Nb of recruited tnf+ macrophages
15
10
5
0
(8)
(8)
DMSO
PP2

Fig. S6

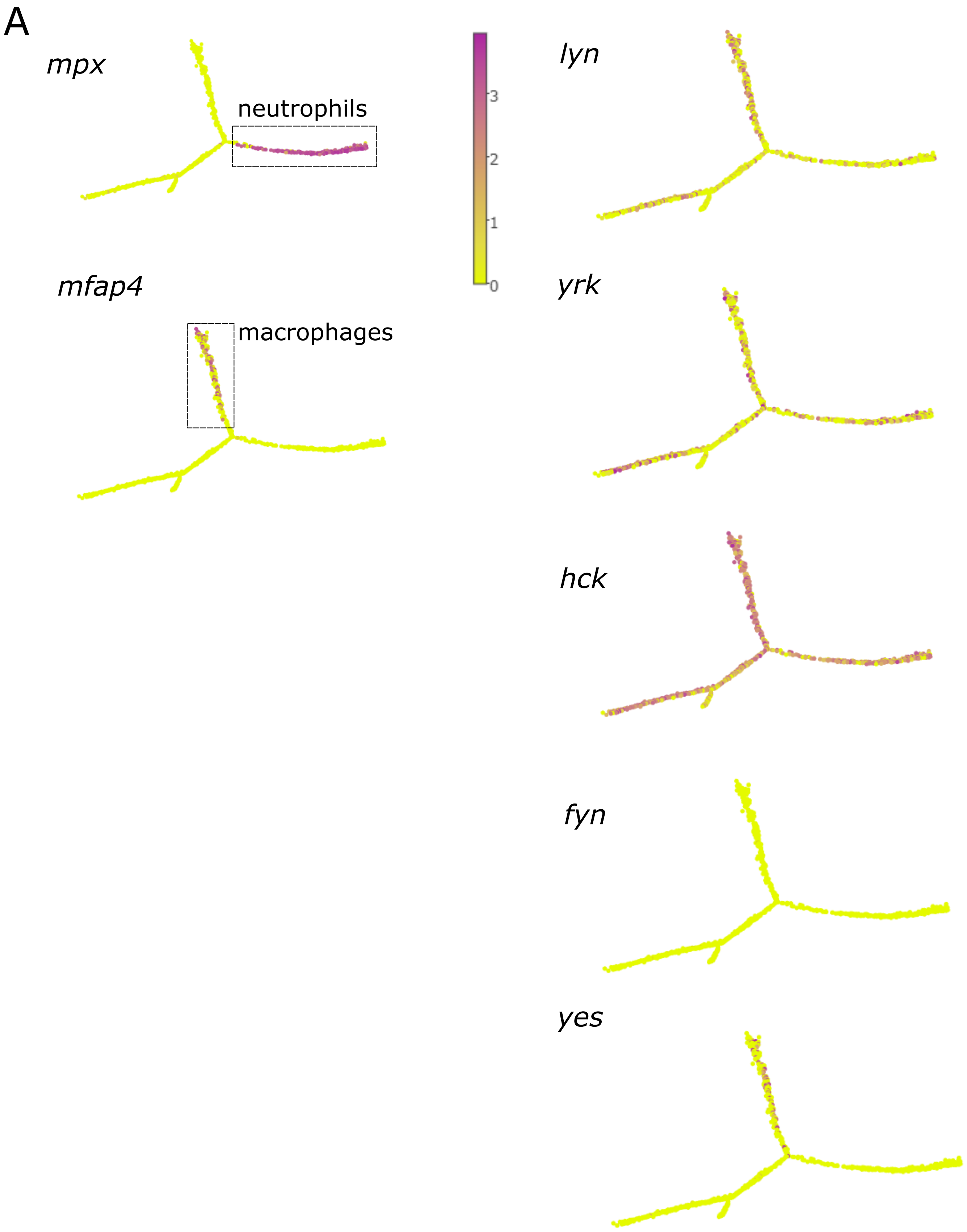
A
mpx
neutrophils
mfap4
macrophages
3
2
1
0
lyn
yrk
hck
fyn
yes

Fig. S7

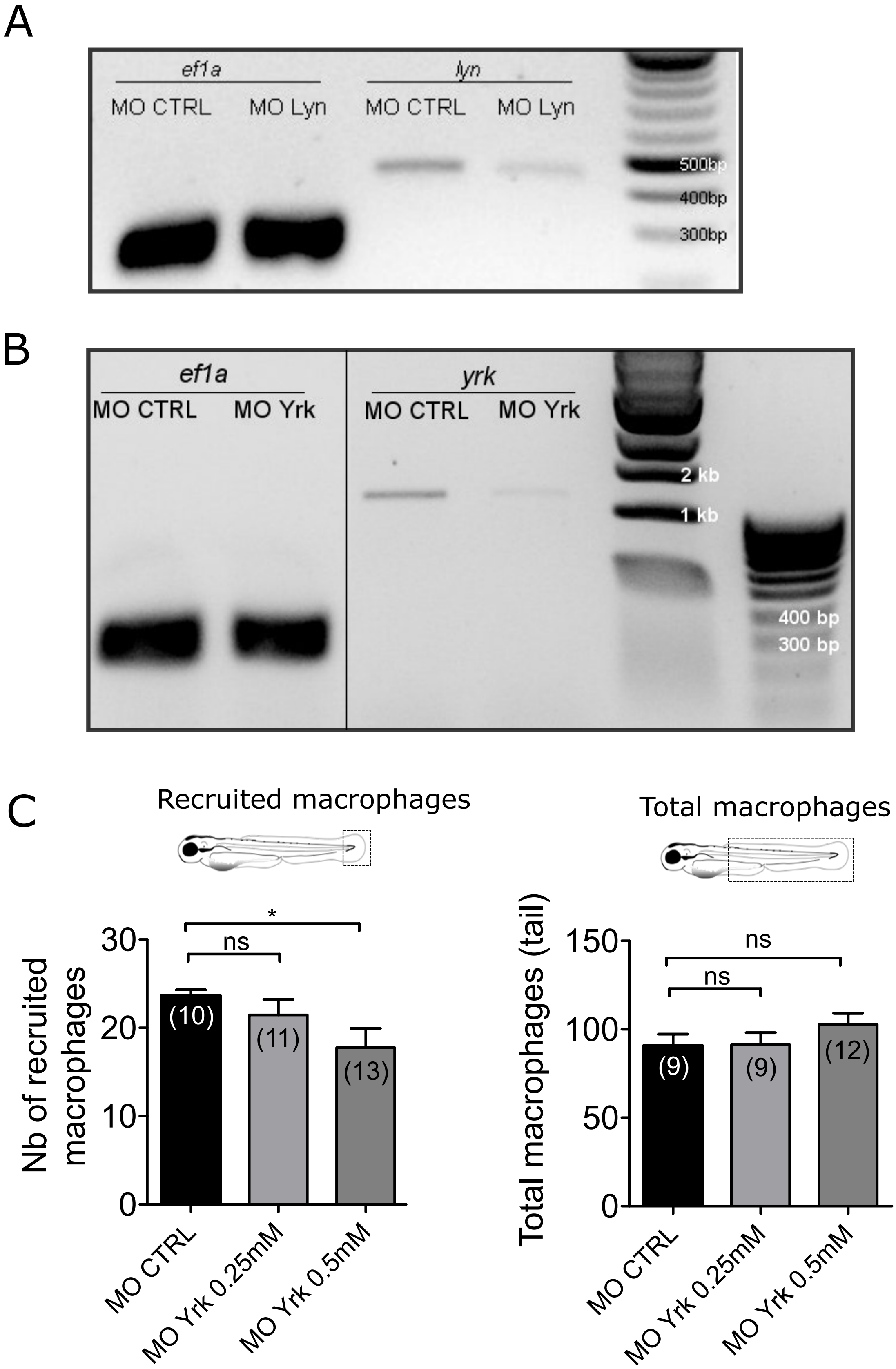
A
ef1a
lyn
MO CTRL
MO Lyn
MO CTRL
MO Lyn
500bp
400bp
300bp
B
ef1a
yrk
MO CTRL
MO Yrk
MO CTRL
MO Yrk
2 kb
1 kb
400 bp
300 bp
C
Recruited macrophages
Total macrophages
Nb of recruited macrophages
Total macrophages (tail)
ns
*
(10)
(11)
(13)
(9)
(9)
(12)
MO CTRL
MO Yrk 0.25mM
MO Yrk 0.5mM

Fig. S8

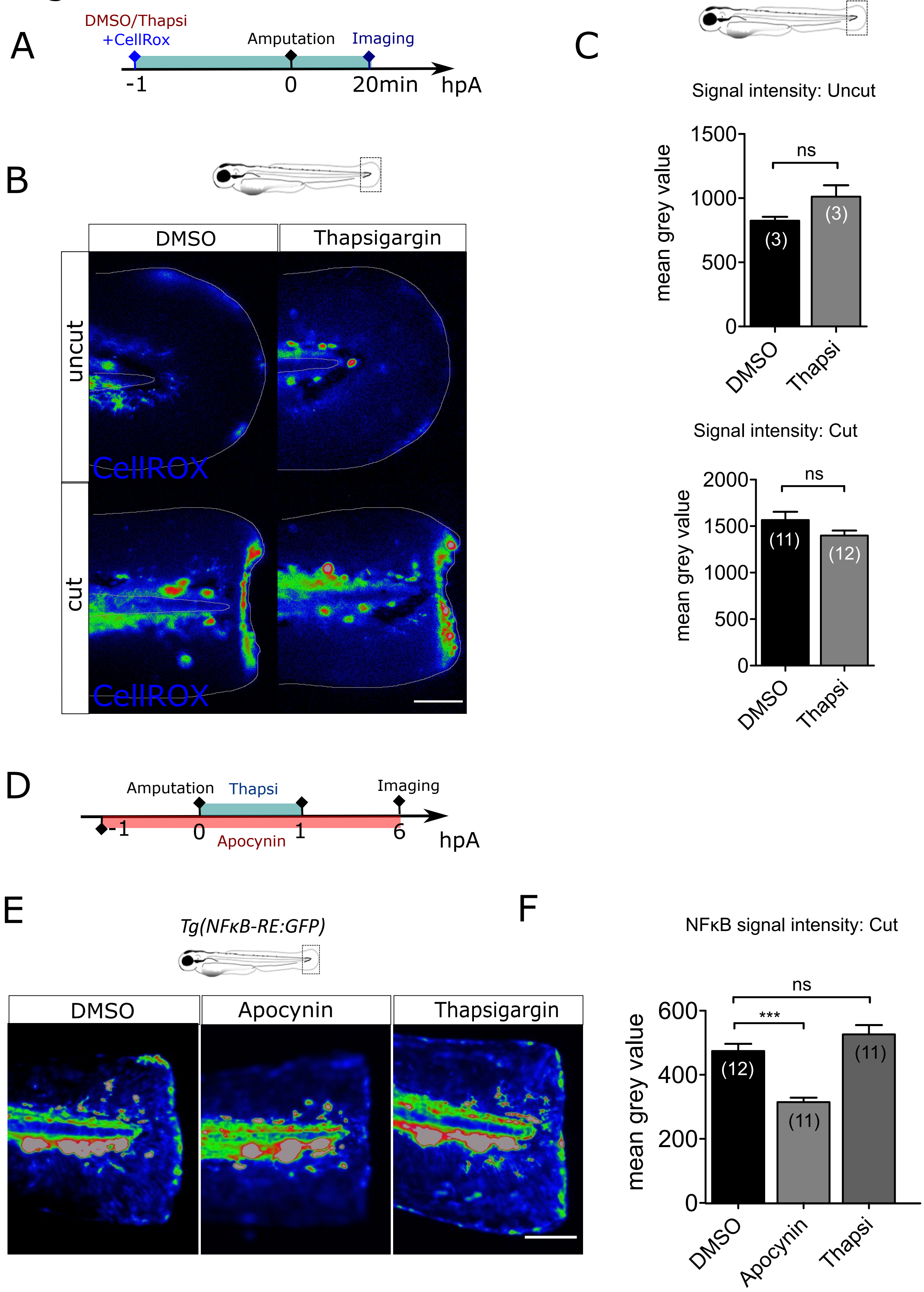
A
DMSO/Thapsi
+CellRox
Amputation
Imaging
-1
0
20min
hpA
B
DMSO
Thapsigargin
uncut
cut
CellROX
CellROX
C
Signal intensity: Uncut
mean grey value
1500
1000
500
0
ns
(3)
(3)
DMSO
Thapsi
Signal intensity: Cut
mean grey value
2000
1500
1000
500
0
ns
(11)
(12)
DMSO
Thapsi
D
Amputation
Thapsi
Imaging
-1
0
1
6
Apocynin
hpA
E
Tg(NFκB-RE:GFP)
DMSO
Apocynin
Thapsigargin
F
NFκB signal intensity: Cut
mean grey value
600
400
200
0
ns
***
(12)
(11)
(11)
DMSO
Apocynin
Thapsi
